# Knockdown resistance (*kdr)* and organochlorine insecticide resistance in malaria vectors: a systematic review

**DOI:** 10.1186/s12936-025-05659-1

**Published:** 2025-11-19

**Authors:** Ebrahim Abbasi, Salman Daliri

**Affiliations:** 1 Department of Biology and Control of Disease Vectors, School of Health, Shiraz University of Medical Sciences, Zand Street, JGHF+XFG, P.O. Box 3761833650, Shiraz, Fars Province Iran; 2 Shiraz University of Medical Sciences, Shiraz, Iran

**Keywords:** Malaria vectors, Knockdown resistance, Organochlorine insecticides, Insecticide resistance, *Anopheles*, Systematic review

## Abstract

**Background:**

*Anopheles albimanus*, *Anopheles darlingi*, *Anopheles dirus*, and *Anopheles punctipennis* are key malaria vectors across multiple regions. Insecticide resistance especially to organochlorines poses a major challenge to vector control. While knockdown resistance (*kdr*) mutations are a well-known resistance mechanism, evidence in these species remains inconclusive. This study systematically reviewed the presence of *kdr* mutations associated with organochlorine resistance in these four *Anopheles* species.

**Methods:**

A systematic review was conducted in accordance with PRISMA guidelines. Six international databases PubMed, Web of Science, Scopus, Cochrane Library, ScienceDirect, and Google Scholar were searched up to June 2024. Keywords included “kdr mutation,” “organochlorine resistance,” “malaria vectors,” and related terms. Inclusion criteria focused on studies examining *kdr* mutations and organochlorine resistance in the four target *Anopheles* species. The quality of studies was assessed using the STROBE checklist. Five studies met all eligibility criteria and were included.

**Results:**

Across the five included studies, no *kdr* mutations (particularly at codons 1010, 1013, or 1014 in the VGSC gene) were identified in any of the four species. Resistance observed in these mosquitoes was attributed primarily to metabolic mechanisms, including elevated activity of mixed-function oxidases (MFO) and non-specific esterases (NSE), rather than target-site insensitivity.

**Conclusion:**

Current evidence indicates that *kdr* mutations do not contribute to organochlorine resistance in *An. albimanus*, *An. darlingi*, *An. dirus*, or *An. punctipennis*. Metabolic and behavioural resistance mechanisms appear to play a larger role. Given the limited number of studies and gene regions analysed, further research with broader genetic screening is essential to fully understand resistance dynamics in these vectors.

**Supplementary Information:**

The online version contains supplementary material available at 10.1186/s12936-025-05659-1.

## Background

*Anopheles* is the primary vector of malaria in the world and the Genus includes more than 400 species, 40 of which are known as malaria vectors. *Anopheles albimanus* is one of the main malaria vectors in the Caribbean islands, Central America, and the northern regions of South America [1]. *Anopheles albimanus* is zoophilic, exophagic, and corpuscular and has higher compatibility than other anopheline species [2]. This species has a flexible behaviour that can affect its spread in lower and more temperate latitudes [3]. Various insecticides, such as organochlorine, organophosphorus, and pyrethroids, have been used to control this vector. However, in recent decades, resistance against the insecticides DDT, lambda-cyhalothrin, deltamethrin, and malathion has been widely reported in this mosquito [4–6]. Various factors have been mentioned as the causes of resistance in this mosquito, but the most important of them are metabolic resistance and target-site resistance, particularly knockdown resistance (*kdr*), which is a form of target-site resistance involving mutations in the voltage-gated sodium channel gene. Although studies have mentioned various results in this field, further investigation is required [7].

*Anopheles darlingi* is one of the main vectors of malaria, and as the dominant species in the Neotropical region, it leads to malaria transmission [8]. This mosquito is widely present in South and Central America, Panama, Mexico, Argentina, and the Atlantic coast [8, 9]. The population of *An. darlingi* increased due to deforestation and human settlements [10, 11]. The species is the focal vector of malaria in South and Central America, causing an increase in malaria in this region [12, 13]. Due to its endophilic properties, this transporter is controlled by the IRS. After the use of DDT to fight *Anopheles* was suspended, organochlorine, organophosphorus, and pyrethroids, including lambda-cyhalothrin and deltamethrin, are used to fight this vector [14]. However, recently, resistance of this vector against insecticides has increased and even led to cross-resistance between insecticides. The existence of cross-resistance among different insecticides, which can be caused by mutations in target sites and metabolic mechanisms, is a serious obstacle to its use in malaria control programmes [15].

*Anopheles dirus* is a malaria vector in Southeast Asia and is common in Thailand, Myanmar, Indochina, and China [16–18]. It has seven different types; the most common types are A, B, C, and D. These species have high biological, behavioural, and biological diversity and remarkable ability to adapt to the environment [16, 19, 20]. This mosquito is prevalent in dense forest areas and is one of the leading causes of falciparum malaria in this region. Behavioural change and their ability to adapt to the environment have increased the resistance of this vector against various types of insecticides [21].

The development of resistance in *Anopheles* mosquitoes has made it very difficult to control these vectors. Mechanisms of resistance to insecticides in *Anopheles* include metabolic resistance, resistance at the target site, cuticular resistance, and behavioural resistance, of which resistance at the target site and metabolic resistance play a significant role in creating resistance to insecticides [22]. Resistance at the target site, with a change in the amino acid sequence of the voltage-gated sodium channel, leads to a decrease in the sensitivity of mosquitoes to pyrethroid and organochlorine insecticides and is known as KDR [23, 24]. The widespread distribution of insecticide resistance, especially organochlorine insecticides worldwide, has affected the epidemiology of malaria. Therefore, raising awareness about the impact of insecticides and the potential for resistance is crucial for understanding and managing malaria transmission. Accordingly, monitoring the resistance of malaria vectors against insecticides to implement control programs is essential to verify the effectiveness of control tools. Thus, this study was conducted to investigate the presence of *kdr* mutation in causing resistance to organochlorine insecticides in *An. albimanus*, *An. darlingi*, *An. dirus* and *An. punctipennis*worldwide using a systematic review method[25, 26].

## Methods

### Study protocol

This study is a systematic review in the field of kdr mutation in *An. albimanus, An. darlingi, An. dirus*, and *An. punctipennis* based on PRISMA guidelines (Preferred Reporting Items for Systematic Reviews and Meta-Analyses) [27]. Also, this study has been registered in the International Prospective Register of Systematic Reviews (PROSPERO) with the code CRD42021231605.

### Search strategy

Articles were extracted without a time limit until the end of June 2024 from PubMed, Web of Science, Cochrane Library, Scopus, Science Direct, and Google Scholar databases and keywords of resistance, knockdown mutations, knockdown resistance, kdr, insecticide resistance, organochlorine insecticide, organochlorine, insecticide, dichloroethane, DDT, aldrin, lindane, dieldrin, malaria vectors, *An. albimanus, An. darlingi, An. dirus*, and *An. punctipennis* were searched in the title, abstract and keywords in singular and compound form using AND and OR operators.

### Inclusion and exclusion criteria

Studies were included if they met all the following criteria: (1) published in English; (2) primary research articles (i.e., not reviews, letters, or conference abstracts); (3) investigated one or more of the four target *Anopheles* species; (4) specifically examined knockdown resistance (*kdr*) mutations; and (5) included data on resistance to at least one organochlorine insecticide (e.g., DDT, dieldrin, aldrin, or lindane). Studies were required to use molecular methods (e.g., gene sequencing) to detect the presence or absence of kdr mutations. Studies were excluded if they met one or more of the following criteria: (1) did not focus on *kdr* mutations; (2) investigated resistance to insecticides other than organochlorines without reporting relevant data on organochlorines; (3) involved species outside of the target *Anopheles* list; or (4) were case reports, case series, or did not present original data (e.g., reviews or editorials). Studies not meeting minimum quality standards based on the STROBE checklist were also excluded.

### Quality assessment

The quality assessment of the articles was done based on 22 parts of the STROBE (Strengthening the Reporting of Observational Studies in Epidemiology) checklist, examining compliance with the principles of writing and implementation in the title, method of reporting findings, limitations, and conclusions. Each item on the checklist is assigned a score based on importance, with a maximum possible score of 33 [28].

### Screening and data extraction

At first, considering the inclusion and exclusion criteria, the title and abstract of the articles were examined independently by two researchers. Two researchers then reviewed the full text of the articles. If an article was rejected, the reason was documented. In cases of disagreement between the two researchers, a third reviewer evaluated the article. Data extraction was done using a previously prepared checklist, including study location, study time, insecticide type, vector type, and *kdr* mutation.

## Results

Globally, about 40 species of *Anopheles* mosquitoes are able to carry malaria parasites. Understanding their biological and behavioural characteristics, as well as effective control methods, is essential for combating and reducing malaria. However, insecticide resistance poses a significant obstacle to controlling these vectors. Organochlorine insecticides have been used to control these vectors for a long time, but resistance, especially of the *kdr* type, has limited the use of these insecticides.

### *kdr* in *Anopheles albimanus*

*Anopheles albimanus* is one of the main malaria vectors in various world regions [3]. In the past, resistance to organochlorine insecticides, which are widely used to deal with this vector, has been reported. Studies of metabolic mechanisms or insensitivity of the target site, such as mutations in the sodium channel gene (VGSC), have mentioned the cause of this resistance [29, 30]. It has been shown that increasing the activity level of esterases and oxidases plays an important role in creating resistance in this vector [31]. High oxidase activity and a target site mechanism have also been implicated in cross-resistance between DDT and pyrethroids in *An. albimanus* [32]. Considering the research done on *kdr* mutation in *An. albimanus*, only one study has been conducted in Colombia by Orjuela *et al.* [46] in which sequencing of all the samples in the forward and reverse direction has been done in the context of three codons of 1010, 1013, and 1014, which are related to resistance to organochlorine insecticides and pyrethroids in malaria vectors. The findings identified the GTT codon at position 1010 (V1010), AAC codon (for asparagine) at position 1013 (N1013), and TTA and TTG codons (for leucine) at position 1014, revealing that no amino acid mutation was observed in the sequences and there is no *kdr* mutation in them [33]. Even though this study has shown that the *kdr* mutation plays a role in *An. albimanus* resistance, it is not resistant to organochlorine insecticides, and more studies are required to confirm this claim.

### *kdr *in *Anopheles darlingi*

*Anopheles darlingi* is one of the vectors of malaria in the world, and it can be found especially in the Amazon region and Africa. This vector has a wide global occurrence and differentiation in genetic, morphological, and behavioural traits, leading to the creation and spread of various insecticide resistance mechanisms [34, 35]. For many years, neurotoxic insecticides have been used to deal with this vector. To deal with this vector, it is necessary to evaluate its sensitivity; however, the resistance or sensitivity of this vector has rarely been evaluated so far. Organochlorine insecticides, especially DDT, cause paralysis or death of this transporter by targeting the voltage-gated sodium channel [29, 36]. However, *kdr* mutation has recently been considered. In the study of Orjuela *et al.* [46] in Colombia done on *An. darlingi*, knockdown mutations did not create resistance to organoleptic insecticides. In this study, all samples were analysed in the forward and reverse direction of sequencing, and three codons, 1010, 1013, and 1014, are related to resistance to organochlorine insecticides and pyrethroids in malaria vectors. The findings identified the GTT codon at position 1010 (V1010), AAC codon (for asparagine) at position 1013 (N1013), and TTA and TTG codons (for leucine) at position 1014, indicating that no amino acid mutation was observed in the sequences and there is no *kdr* in them [33]. Fonseca-González *et al.* in Colombia investigated *kdr* in *An. darlingi* against organochlorine insecticides and pyrethroids. The findings showed that all populations of *An. darlingi* were sensitive to deltamethrin, permethrin, and malathion. Resistance to lambda-cyhalothrin and DDT was observed in the population, demonstrating 65 to 75% mortality. Cross-resistance between these two insecticides was also observed. However, specific resistance due to *kdr* was not observed, and the cause of resistance was reported to be increased metabolism through MFO and NSE [6]. Based on the investigated studies, it can be mentioned that *kdr* mutation has not been observed in *An. darlingi*, and the cause of resistance in this vector can be metabolic mechanisms and increased activity of oxidases and esterases.

### *kdr *in *Anopheles dirus*

*Anopheles dirus* complex is one of the malaria vectors that can be found in Asia, especially its forest areas. This complex has biological and ecological differences and behavioural changes when it is in contact with humans and exposed to environmental stimuli which can improve carrying capacity and environmental adaptation [16, 37, 38]. These changes can increase resistance to control measures in this mosquito Organochlorine insecticides have been one of the main insecticides used to combat this vector since a long time ago, and the main cause of resistance to these insecticides was reported to be *kdr* mutation [39]. Verhaeghen *et al.* investigated *kdr* in 73 *An. dirus* mosquitoes in Vietnam. Based on their study, sequencing was performed in the DIIS6 region of the para-type sodium channel gene for the samples to identify *kdr*, but the results did not show any *kdr* mutations among the samples [40]. Sumarnrote *et al.* [41] investigated the *kdr* mutation in *An. dirus* against the insecticide deltamethrin in Vietnam. The results of the genetic sequencing of the samples did not show any L1014F or L1014S substitutions in the VGSC gene.

Additionally, the attenuation ratio of this transporter against deltamethrin was 100%, which indicates that it is sensitive to this insecticide. In the study by Zeng *et al.* in Hainan Province, the attenuation ratio of *An. dirus* against the insecticides deltamethrin (0.05%), DDT (4%), malathion (5%), and cyfluthrin (0.15%) was 100%, revealing the high sensitivity of this vector against organochlorine insecticides. It should also be mentioned that no *kdr* mutation was observed in this study [21]. In general, *kdr* mutation has not been detected to create resistance in this vector, and this vector has a high sensitivity to insecticides, including organochlorines.

### *kdr* in *Anopheles punctipennis*

**Figure 1:**
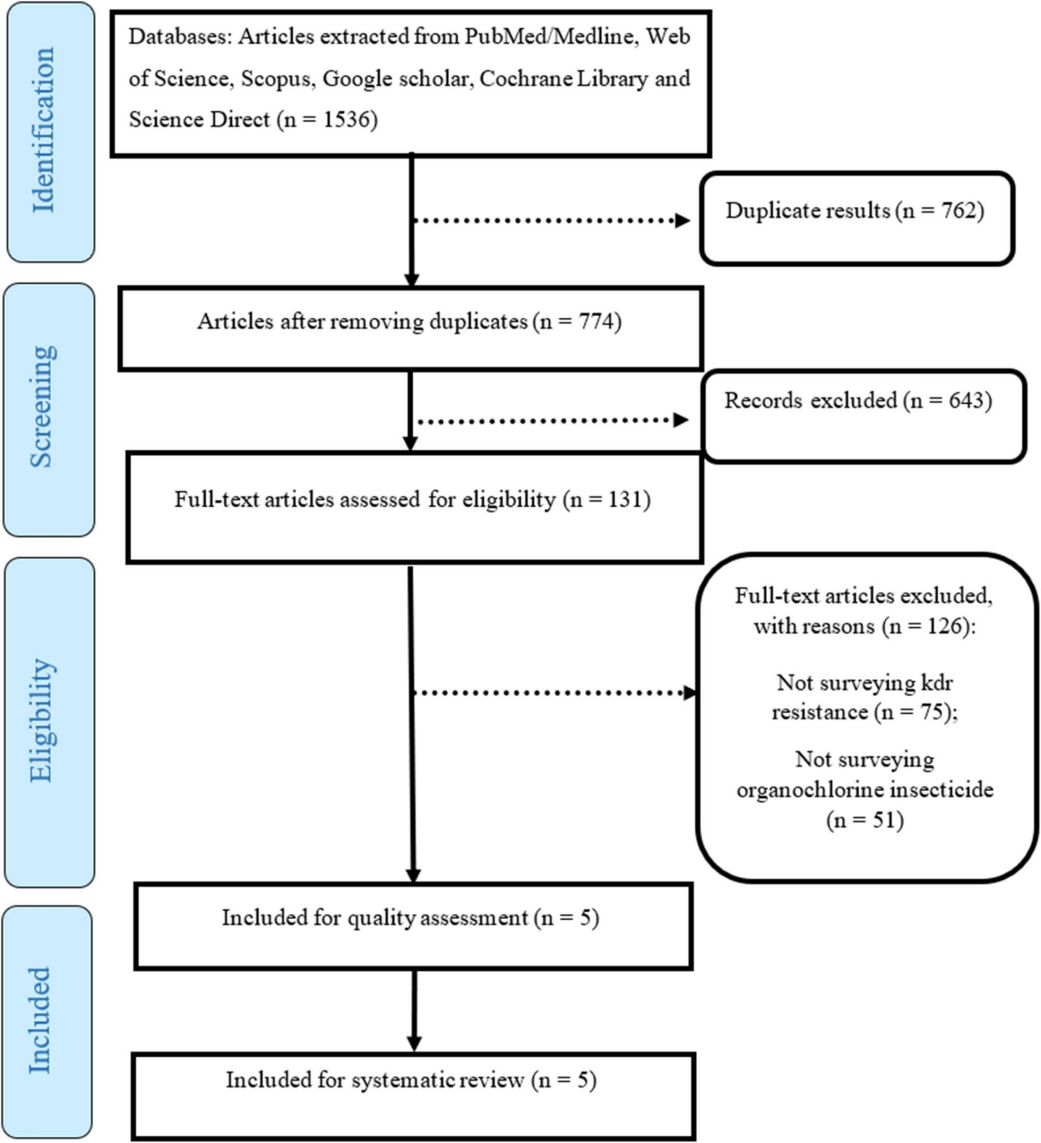
The review process based on the PRISMA flow diagram

*Anopheles punctipennis* is a common and widespread malaria vector in the United States and North America [3]. This vector can transmit *Plasmodium vivax* and *Plasmodium falciparum* [9]. It can also transmit *Plasmodium odocoilei* to wild animals such as deer, which increases their biodiversity and makes fighting it more complex [42, 43]. Also, creating resistance to insecticides in this vector can be another limitation of controlling it. While searching, only one study was found which investigated the *kdr* mutation in this vector. Orjuela *et al.* investigated the *kdr* mutation in *An. punctipennis*. The results of sequencing the samples identified the GTT codon at position 1010 (V1010), AAC codon at position 1013 (N1013), and TTA and TTG codons at position 1014. which shows that no amino acid and *kdr* mutations were observed in the sequences [33]. Although this study has shown no *kdr* mutation in this vector, more studies are needed to confirm this issue (Table 1 and Fig. 1).

**Table 1:** Characteristics of the articles included in the systematic review

Author	Year study	Place study	Sample size	Type of *Anopheles*
Orjuela L [33]	2019	Colombia	126	*An. albimanus, An. Darling* and *An. Punctipennis*
Fonseca-González I [6]	2009	Colombia	120	*An. darlingi*
Verhaeghen K [40]	2009	Mekong region	60	*An. dirus*
Sumarnrote A [41]	2017	Thailand	34	*An. dirus*
Zeng LH [21]	2011	China	90	*An. dirus*

## Discussion

In this study, *kdr* resistance in *An. albimanus* was investigated, and the findings showed no *kdr* resistance mutation in this vector. There are various mechanisms for *Anopheles’* insecticide resistance, including behavioural, cuticular, metabolic, and target site resistance, in which metabolic resistance and resistance to the target site play a greater role in creating resistance to insecticides [44]. In the studies that were conducted, it was mentioned that metabolic resistance creates resistance in *An. albimanus*. In sodium channel 9, natural mutations at positions N1013 (S), V1010 (L), I1048 (N), L1014 (F/S/C/W), N1575 (Y) and S1156 (G) are found to be associated with the phenotype resistance in *Anopheles* [6, 45]. Besides, only mutations identified at position 1014 are involved in reducing sodium channel sensitivity to insecticides [7, 46]. *Anopheles albimanus* mutations at positions L1014F and L1014C can lead to *kdr*, which has not been identified in the studies carried out so far [32]. However, it should also be noted that only a small region of the VGSC gene is often evaluated in sequencing. As a result, there is a possibility that mutations and resistance created by mutations occurring in different regions of the gene can be communicated, so it is recommended to examine a longer region of the gene [7]. In general, the studies reporting *kdr* mutation's role in creating resistance in *An. albimanus* have not been provided. However, confirmation of this issue requires conducting more studies in this field.

While this review clearly establishes the absence of *kdr* mutations in *An. albimanus*, *An. darlingi*, *An. dirus*, and *An. punctipennis*, a more comprehensive discussion of the underlying limitations and broader implications is warranted. Most of the studies included in this review focused only on a small fragment of the VGSC gene, typically around codons 1010, 1013, and 1014. This limited scope may overlook other potential mutations elsewhere in the gene that could contribute to insecticide resistance. Future studies employing whole-genome sequencing (WGS), RNA-Seq transcriptomic analysis, or broader molecular screening could provide a more complete understanding of resistance mechanisms. Additionally, environmental and behavioural factors, such as vector feeding preferences, exophilic tendencies, and habitat variation, may contribute significantly to reduced insecticide efficacy even in the absence of target-site mutations. These interactions between vector biology, ecology, and resistance should be explored in future research. Lastly, the absence of *kdr* mutations in these species, despite widespread organochlorine usage in many endemic regions, suggests either a true lack of selection pressure or a current gap in surveillance and molecular diagnostic coverage. Addressing these gaps with standardized, cross-regional studies would greatly enhance the understanding of resistance evolution and support more effective vector control strategies.

This systematic review was conducted using a comprehensive and structured approach; however, several limitations should be acknowledged. One major limitation lies in the limited genomic scope of the included studies. Most focused only on a small portion of the voltage-gated sodium channel (VGSC) gene, particularly codons 1010, 1013, and 1014. As a result, other potentially relevant mutations elsewhere in the gene may have been overlooked. In terms of geographic representation, the studies reviewed were primarily conducted in specific regions such as Colombia, Southeast Asia, and North America. This regional concentration limits the generalizability of the findings to other malaria-endemic areas, including Sub-Saharan Africa and the Western Pacific. Another important issue is the potential for language and publication bias. Only English-language articles were included, and relevant studies published in other languages or within grey literature may have been inadvertently excluded. This could result in selection bias. Additionally, although all included studies met the basic quality criteria based on the STROBE checklist, there was variation in methodological quality, vector populations studied, and molecular protocols used. Such heterogeneity reduces the comparability of results across studies. The review also faced challenges related to data synthesis. Due to substantial variation in study designs and reporting formats, performing a quantitative synthesis or meta-analysis was not feasible, and findings were instead synthesized qualitatively. Furthermore, despite independent screening by multiple reviewers, subjective decisions regarding inclusion and data interpretation could have introduced reviewer bias. Lastly, some full-text articles could not be accessed or lacked sufficient methodological detail, which may have resulted in the exclusion of studies that were otherwise relevant based on keyword screening. Despite these limitations, the findings of this review contribute meaningful insights into the current understanding of insecticide resistance mechanisms and underscore the need for broader, standardized genetic surveillance in malaria vector research.

In the present systematic review*, kdr* resistance in *An. darlingi* and *An. dirus* was not reported. Other studies have shown that in the populations of *An. darlingi* and *An. dirus*, metabolic mechanisms have played an essential role in resistance, and the level of functional oxidases has increased in resistant *Anopheles* [6]. Additionally, in pyrethroid-resistant *Anopheles*, an increase in the levels of non-specific esterases has been reported [45]. In vectors resistant to organochlorine insecticides and pyrethroids, metabolic detoxification and reduction of cuticular penetration have also been observed [47–51]. These cases can generally cause high resistance levels without *kdr* mutations [52]. Other studies investigating the resistance of *Anopheles An. darlingi* has reported high levels of AChE against organophosphates and carbamates [31]. In a study conducted in Mexico, an increase in the level of cytochrome P450 and the activity of glutathione S-transferase and esterase was observed in DDT-resistant *Anopheles* [31]. Studies have shown that in the population of *An. darlingi*, resistance to carbamates and organophosphates is associated with mutations in *ace-1*, resistance to pyrethroids is associated with mutations in the VGSC gene, and resistance to deltamethrin and alpha-cypermethrin is associated with cytochrome P450 CYP9K1 and overexpression of CYP6P5 [32, 53, 54].

Considering the presence of *kdr* mutations in most DDT-resistant *Anopheles* species in different regions of the world, the absence of this mutation in the mentioned vectors can reflect genetic limitations or reduce insecticide pressure [53, 55]. Also, the activity of *An. dirus* is strongly related to the foothills, deep forests, and forest margins, which can affect its behavioural characteristics and reduce the effect of insecticides on them [16]. Since no *kdr* mutation was observed in the mentioned vectors, metabolic and behavioural resistance mechanisms can play a major role in developing resistance. Hence, Various factors and mechanisms are likely involved in the development of resistance in *An. darlingi* and *An. dirus*. Overall, the studies have not implicated the *kdr* mechanism in developing resistance. However, studies in this field are few, and more studies are needed.

Although this review provides detailed insight into the role of *kdr* mutations in *Anopheles albimanus*, *An. darlingi*, *An. dirus*, and *An. punctipennis*, the narrow taxonomic focus inherently limits the generalizability of the findings. Many other *Anopheles* species or species complexes such as *Anopheles gambiae*, *Anopheles funestus*, and *Anopheles stephensi* are major malaria vectors in other regions, and their resistance mechanisms may differ significantly due to ecological, genetic, and insecticide exposure differences. Therefore, caution should be exercised in extrapolating these results to other vector populations without supporting molecular evidence. Future research should expand the scope of analysis to include a wider range of species and geographical areas to build a more comprehensive understanding of *kdr* mutation dynamics and resistance patterns globally.

## Conclusion

Based on the findings, *kdr* mutation does not play any role in creating resistance in *An. albimanus*, *An. darlingi, An. dirus,* and *An. punctipennis*. Considering the diversity of behavioural and biological characteristics of these mosquitoes, other metabolic and behavioural causes can play a role in creating resistance against organochlorine insecticides. Besides, limited studies have been done to investigate the resistance of *kdr* in these transporters; as a result, it is necessary to conduct more studies on the activity of these transporters in the field of resistance-causing factors.

## Supplementary Information

Supplementary material 1

## Data Availability

All data obtained from this study are included in the text of article.
