# Supplementary material for: Knockdown resistance (kdr) and organochlorine insecticide resistance in malaria vectors: a systematic review
Source: Malar J. 2025 Nov 19;24:415. doi: 10.1186/s12936-025-05659-1 (PMC12628609; doi:10.1186/s12936-025-05659-1)
Supplement: Supplementary file 1 — Supplementary material 1 [file 12936_2025_5659_MOESM1_ESM.docx]

| **Section and Topic** | **Item #** | **Checklist item** | **Location where item is reported** |
| --- | --- | --- | --- |
| **TITLE** | | |  |
| Title | 1 | Identify the report as a systematic review. | Page 1 |
| **ABSTRACT** | | |  |
| Abstract | 2 | See the PRISMA 2020 for Abstracts checklist. | Page 2 |
| **INTRODUCTION** | | |  |
| Rationale | 3 | Describe the rationale for the review in the context of existing knowledge. | Page 3 |
| Objectives | 4 | Provide an explicit statement of the objective(s) or question(s) the review addresses. | Page 3 |
| **METHODS** | | |  |
| Eligibility criteria | 5 | Specify the inclusion and exclusion criteria for the review and how studies were grouped for the syntheses. | Page 4 |
| Information sources | 6 | Specify all databases, registers, websites, organisations, reference lists and other sources searched or consulted to identify studies. Specify the date when each source was last searched or consulted. | Page 4 |
| Search strategy | 7 | Present the full search strategies for all databases, registers and websites, including any filters and limits used. | Page 4 |
| Selection process | 8 | Specify the methods used to decide whether a study met the inclusion criteria of the review, including how many reviewers screened each record and each report retrieved, whether they worked independently, and if applicable, details of automation tools used in the process. | Page 4 |
| Data collection process | 9 | Specify the methods used to collect data from reports, including how many reviewers collected data from each report, whether they worked independently, any processes for obtaining or confirming data from study investigators, and if applicable, details of automation tools used in the process. | Page 4 |
| Data items | 10a | List and define all outcomes for which data were sought. Specify whether all results that were compatible with each outcome domain in each study were sought (e.g. for all measures, time points, analyses), and if not, the methods used to decide which results to collect. | Page 4 |
|  | 10b | List and define all other variables for which data were sought (e.g. participant and intervention characteristics, funding sources). Describe any assumptions made about any missing or unclear information. | Page 4 |
| Study risk of bias assessment | 11 | Specify the methods used to assess risk of bias in the included studies, including details of the tool(s) used, how many reviewers assessed each study and whether they worked independently, and if applicable, details of automation tools used in the process. | Page 4 |
| Effect measures | 12 | Specify for each outcome the effect measure(s) (e.g. risk ratio, mean difference) used in the synthesis or presentation of results. | Page 4 |
| Synthesis methods | 13a | Describe the processes used to decide which studies were eligible for each synthesis (e.g. tabulating the study intervention characteristics and comparing against the planned groups for each synthesis (item #5)). | Page 4 |
|  | 13b | Describe any methods required to prepare the data for presentation or synthesis, such as handling of missing summary statistics, or data conversions. | Page 4 |
|  | 13c | Describe any methods used to tabulate or visually display results of individual studies and syntheses. | Page 4 |
|  | 13d | Describe any methods used to synthesize results and provide a rationale for the choice(s). If meta-analysis was performed, describe the model(s), method(s) to identify the presence and extent of statistical heterogeneity, and software package(s) used. | Page 4 |
|  | 13e | Describe any methods used to explore possible causes of heterogeneity among study results (e.g. subgroup analysis, meta-regression). | Page 4 |
|  | 13f | Describe any sensitivity analyses conducted to assess robustness of the synthesized results. | Page 4 |
| Reporting bias assessment | 14 | Describe any methods used to assess risk of bias due to missing results in a synthesis (arising from reporting biases). | Page 4 |
| Certainty assessment | 15 | Describe any methods used to assess certainty (or confidence) in the body of evidence for an outcome. | Page 4 |
| **RESULTS** | | |  |
| Study selection | 16a | Describe the results of the search and selection process, from the number of records identified in the search to the number of studies included in the review, ideally using a flow diagram. | Page 4 and 5 |
|  | 16b | Cite studies that might appear to meet the inclusion criteria, but which were excluded, and explain why they were excluded. | Page 4 and 5 |
| Study characteristics | 17 | Cite each included study and present its characteristics. | Page 5 |
| Risk of bias in studies | 18 | Present assessments of risk of bias for each included study. | Page 5 |
| Results of individual studies | 19 | For all outcomes, present, for each study: (a) summary statistics for each group (where appropriate) and (b) an effect estimate and its precision (e.g. confidence/credible interval), ideally using structured tables or plots. | Page 6 |
| Results of syntheses | 20a | For each synthesis, briefly summarise the characteristics and risk of bias among contributing studies. | Page 4-6 |
|  | 20b | Present results of all statistical syntheses conducted. If meta-analysis was done, present for each the summary estimate and its precision (e.g. confidence/credible interval) and measures of statistical heterogeneity. If comparing groups, describe the direction of the effect. | Page 4-6 |
|  | 20c | Present results of all investigations of possible causes of heterogeneity among study results. | Page 4-6 |
|  | 20d | Present results of all sensitivity analyses conducted to assess the robustness of the synthesized results. | Page 4-6 |
| Reporting biases | 21 | Present assessments of risk of bias due to missing results (arising from reporting biases) for each synthesis assessed. | Page 4-6 |
| Certainty of evidence | 22 | Present assessments of certainty (or confidence) in the body of evidence for each outcome assessed. | Page 4-6 |
| **DISCUSSION** | | |  |
| Discussion | 23a | Provide a general interpretation of the results in the context of other evidence. | Page 6-7 |
|  | 23b | Discuss any limitations of the evidence included in the review. | Page 6-7 |
|  | 23c | Discuss any limitations of the review processes used. | Page 6-7 |
|  | 23d | Discuss implications of the results for practice, policy, and future research. | Page 6-7 |
| **OTHER INFORMATION** | | |  |
| Registration and protocol | 24a | Provide registration information for the review, including register name and registration number, or state that the review was not registered. | Page 4 |
|  | 24b | Indicate where the review protocol can be accessed, or state that a protocol was not prepared. | Page 4 |
|  | 24c | Describe and explain any amendments to information provided at registration or in the protocol. | Page 4 |
| Support | 25 | Describe sources of financial or non-financial support for the review, and the role of the funders or sponsors in the review. | Page 7-8 |
| Competing interests | 26 | Declare any competing interests of review authors. | Page 7-8 |
| Availability of data, code and other materials | 27 | Report which of the following are publicly available and where they can be found: template data collection forms; data extracted from included studies; data used for all analyses; analytic code; any other materials used in the review. | Page 7-8 |

*From:*  Page MJ, McKenzie JE, Bossuyt PM, Boutron I, Hoffmann TC, Mulrow CD, et al. The PRISMA 2020 statement: an updated guideline for reporting systematic reviews. BMJ 2021;372:n71. doi: 10.1136/bmj.n71

**Knockdown Resistance (kdr) Associated Organochlorine Resistance in Mosquito-Borne Diseases (*Anopheles albimanus, Anopheles darlingi, Anopheles dirus* and *Anopheles punctipennis*): A Systematic Review Study**

Pubmed/MEDLINE

(“knockdown resistance”[all] OR (knockdown[all] AND resistance[all]) OR KDR[all]) AND ("Organochlorine insecticide"[ALL] OR (Organochlorine[ALL] AND insecticide[ALL]) OR "chlorinated insecticide"[ALL] OR "insecticide organochlorine"[ALL] OR "insecticides organochlorine"[ALL] OR (dichloro[ALL] AND bis[ALL]) OR (chlorophenyl[ALL] AND ethane[ALL]) OR (bis[ALL] AND chlorophenyl[ALL]) OR dichloroethane[ALL] OR (para[ALL] AND chlorophenyl[ALL]) OR (bis[ALL] AND dichloroethane[ALL]) OR (dichloro[ALL] AND ethane[ALL]) OR (para[ALL] AND ethylphenyl[ALL]) OR (dichloroethylidene[ALL] AND chlorobenzene[ALL]) OR parachlorophenyl[ALL] OR dichloroethane[ALL] OR chlorophenyl[ALL] OR dichloroethane[ALL] OR "p chlorophenyl"[ALL] OR dichloroethane[ALL] OR dichlloroethane[ALL] OR dichlorethane[ALL] OR dichloroethane[ALL] OR "dichloroethane analog"[ALL] OR parachlorophenyl[ALL] OR dichloroethane[ALL] OR dichlorodiphenyldichloroethane[ALL] OR DDD[ALL] OR (dichlorodiphenyl[ALL] AND dichloroethane[ALL]) OR dichlorodiphenyldichloroethane[ALL] OR dichlorodiphenyldichloroethane[ALL] OR ddd[ALL] OR (para[ALL] AND ddd[ALL]) OR tde[ALL] OR dichloro[ALL] OR (chlorophenyl[ALL] AND ethylene[ALL]) OR (chlorophenyl[ALL] AND dichloroethylene[ALL]) OR dichloroethylene[ALL] OR (para'[ALL] AND chlorophenyl[ALL]) OR ethylene[ALL] OR (chlorophenyl[ALL] AND ethylene[ALL]) OR (dichloroethenylidene[ALL] AND chlorobenzene[ALL]) OR DDE[ALL] OR (dichlorodiphenyl AND dichloroethylene[ALL]) OR (para[ALL] AND dde[ALL]) OR trichloro[ALL] OR chlorophenyl[ALL] OR (chlorophenyl[ALL] AND chlorophenyl[ALL]) OR trichloroethane[ALL] OR (ortho[ALL] AND chlorophenyl[ALL]) OR "op ddt"[ALL] OR "ortho ddt"[ALL] OR (ortho[ALL] AND dichlorobenzene[ALL]) OR dichlorobenzene[ALL] OR Paradichlorobenzene[ALL] OR "di chloricide"[ALL] OR dichloricide[ALL] OR (para[ALL] AND dichlorbenzene[ALL]) OR (dichloro[ALL] AND benzene[ALL]) OR (para[ALL] AND dichlorobenzene[ALL]) OR paramoth[ALL] OR Aldrin[ALL] OR Isodrin[ALL] OR (hexachloro[ALL] AND hexahydro[ALL]) OR dimethanonaphthalene[ALL] OR (hexachloro[ALL] AND hexahydro[ALL]) OR aldrex[ALL] OR "compound 118"[ALL] OR octalene[ALL] OR photoaldrin[ALL] OR (alpha[ALL] AND hexachlorocyclohexane[ALL]) OR "alpha HCH"[ALL] OR HCH[ALL] OR (benzene[ALL] AND hexachloride[ALL]) OR (alpha[ALL] AND benzenehexachlor[ALL]) OR (alpha[ALL] AND benzenehexachloride[ALL]) OR "alpha bhc"[ALL] OR "alpha hch"[ALL] OR (beta[ALL] AND hexachlorocyclohexane[ALL]) OR cyclohexane[ALL] OR (hexachloro[ALL] AND beta[ALL]) OR (beta[ALL] AND hexachlorobenzene[ALL]) OR (beta[ALL] AND HCH[ALL]) OR "beta Lindane"[ALL] OR (beta[ALL] AND Lindane[ALL]) OR "beta benzene"[ALL] OR (beta[ALL] AND benzene[ALL]) OR hexachloride[ALL] OR "beta hexachloran"[ALL] OR (beta[ALL] AND hexachloran[ALL]) OR "beta 666"[ALL] OR "epsilon HCH"[ALL] OR "beta benzenehexachloride"[ALL] OR "beta bhc"[ALL] OR "beta hch"[ALL] OR campheclor[ALL] OR alltox[ALL] OR camfeclor[ALL] OR camphechlor[ALL] OR "camphene chlorinated"[ALL] OR "chlorinated camphene"[ALL] OR "chlorphen clorphen"[ALL] OR estonox[ALL] OR geniphene[ALL] OR penfene[ALL] OR phenacide[ALL] OR phenatox[ALL] OR phenphene[ALL] OR phenphere[ALL] OR pinetox[ALL] OR polychlorocamphene[ALL] OR toxakil[ALL] OR toxaphen[ALL] OR toxaphene[ALL] OR chlordane[ALL] OR "gamma Chlordane"[ALL] OR Chlordane[ALL] OR (octachloro[ALL] AND alpha[ALL]) OR (alpha[ALL] AND hexahydro[ALL]) OR methanoindene[ALL] OR (octachloro[ALL] AND alpha[ALL]) OR (alpha[ALL] AND tetrahydro[ALL]) OR methanoindan[ALL] OR (octachloro[ALL] AND methane[ALL]) OR (alpha[ALL] AND tetrahydroindane[ALL]) OR (alpha[ALL] AND chlordane[ALL]) OR alphachlordane[ALL] OR "cd 68"[ALL] OR cd68[ALL] OR chlordane[ALL] OR (cis[ALL] AND chlordane[ALL]) OR (cis[ALL] AND photochlordane[ALL]) OR "compound 1068"[ALL] OR "hcs 3260"[ALL] OR "m 410"[ALL] OR "octa klor"[ALL] OR (cis[ALL] AND chlordane[ALL]) OR photochlordane[ALL] OR toxichlor[ALL] OR (trans[ALL] AND chlordane[ALL]) OR "velsicol 1068"[ALL] OR wydane[ALL] OR chlordecone[ALL] OR Kepone[ALL] OR decachlorooctahydro[ALL] OR "metheno cyclobuta"[ALL] OR "pentalen one"[ALL] OR "cg 1189"[ALL] OR cg1189[ALL] OR kepone[ALL] OR chlorphenotane[ALL] OR chlorobenzene[ALL] OR trichloroethylidene[ALL] OR (chlorophenyl[ALL] AND trichloroethane[ALL]) OR trichloroethane[ALL] OR trichloroethene[ALL] OR benzochloryl[ALL] OR chlorophenoltane[ALL] OR chlorophenotane[ALL] OR chlorophenothane[ALL] OR chlorphenethanum[ALL] OR chlorphenotan[ALL] OR chlorphenothane[ALL] OR chlorphenothanum[ALL] OR clofenotan[ALL] OR clofenotane[ALL] OR d.d.t[ALL] OR DDT[ALL] OR "ddt residue"[ALL] OR detane[ALL] OR dichlordiphenyltrichlormethylmethane[ALL] OR dichlordiphenyltrichloroethane[ALL] OR dichlorodiphenyltrichloroethane[ALL] OR dicophane[ALL] OR dodat[ALL] OR esoderm[ALL] OR estonate[ALL] OR gesapon[ALL] OR gesarex[ALL] OR gesarol[ALL] OR lentinol[ALL] OR neocide[ALL] OR "para ddt"[ALL] OR parachlorocide[ALL] OR pentachlorin[ALL] OR penticidum[ALL] OR pestanal[ALL] OR suleo[ALL] OR ethane[ALL] OR trichomon[ALL] OR clofentezine[ALL] OR bisclofentezin[ALL] OR (chlorophenyl[ALL] AND tetrazine[ALL]) OR tetrazine[ALL] OR dieldrin[ALL] OR "Alvit 55"[ALL] OR Alvit55[ALL] OR (hexachloro[ALL] AND epoxy[ALL]) OR "octahydro endo"[ALL] OR "10 para"[ALL] OR "meta dieldrin"[ALL] OR "compound 497"[ALL] OR (dieldrin[ALL] AND abavit[ALL]) OR dieldrine[ALL] OR heod[ALL] OR photodieldrin[ALL] OR endosulfan[ALL] OR Thiodan[ALL] OR Thiodon[ALL] OR (beta[ALL] AND Endosulfan[ALL]) OR Thiotox[ALL] OR (alpha[ALL] AND Endosulfan[ALL]) OR (hexachloronorbornene[ALL] AND sulfite[ALL]) OR (hydroxymethyl[ALL] AND hexachloronorbornenesulfite[ALL]) OR (hydroxymethyl[ALL] AND hexachlorobicyclo[ALL]) OR (ene[ALL] AND sulfite[ALL]) OR (hydroxymethyl[ALL] AND hexachloronorcamphene[ALL]) OR (alpha[ALL] AND benzoepin[ALL]) OR (alpha[ALL] AND endosulfan[ALL]) OR (alpha[ALL] AND endosulfane[ALL]) OR benzoepin[ALL] OR beosit[ALL] OR (beta[ALL] AND benzoepin[ALL]) OR (beta[ALL] AND endosulfan[ALL]) OR (beta[ALL] AND endosulfane[ALL]) OR chlorothiepine[ALL] OR chlorthiapinum[ALL] OR chlorthiepin[ALL] OR cyclodan[ALL] OR endogan[ALL] OR ensawan[ALL] OR "fmc 5462"[ALL] OR fmc5462[ALL] OR (hexachlorohexahydro[ALL] AND methano[ALL]) OR (benzodioxathiepine[ALL] AND oxide[ALL]) OR hexachloronorbornene[ALL] OR (oxymethylene[ALL] AND sulfite[ALL]) OR malix[ALL] OR sialan[ALL] OR thifor[ALL] OR thimul[ALL] OR thiodan[ALL] OR thionex[ALL] OR thiotox[ALL] OR thyodan[ALL] OR thyonex[ALL] OR tiodan[ALL] OR endrin[ALL] OR Hexadrin[ALL] OR "compound 269"[ALL] OR (compound[ALL] AND dieldrin[ALL]) OR hexadrin[ALL] OR heptachlor[ALL] OR (heptachloro[ALL] AND tetrahydro[ALL]) OR methanoindene[ALL] OR heptachlore[ALL] OR heptachloro[ALL] OR (tetrahydro[ALL] AND methanoindene[ALL]) OR photoheptachlor[ALL] OR (heptachlor[ALL] AND epoxide[ALL]) OR (Epoxide[ALL] AND Heptachlor[ALL]) OR Heptachlorepoxide[ALL] OR isobenzan[ALL] OR (octachloro[ALL] AND endomethylene[ALL]) OR tetrahydrophthalan[ALL] OR octachloro[ALL] OR (hexahydro[ALL] AND methanoisobenzofuran[ALL]) OR (hexahydro[ALL] AND methanoisobenzofuran[ALL]) OR (octachloro[ALL] AND hexahydro[ALL]) OR methanoisobenzofuran[ALL] OR (betahydro[ALL] AND methanophthalan[ALL]) OR izobenzan[ALL] OR omtan[ALL] OR ontan[ALL] OR "r 6700"[ALL] OR "sd 4402"[ALL] OR telodrin[ALL] OR lindane[ALL] OR Hexachlorane[ALL] OR (Benzene[ALL] AND Hexachloride[ALL]) OR (Hexachloride[ALL] AND Benzene[ALL]) OR (Epsilon[ALL] AND hexachlorocyclohexane[ALL]) OR (Epsilon[ALL] AND hexachlorocyclohexane[ALL]) OR (Zeta[ALL] AND hexachlorocyclohexane[ALL]) OR (Zeta[ALL] AND hexachlorocyclohexane[ALL]) OR Lindane[ALL] OR (Benzene[ALL] AND Hexachloride[ALL]) OR (Hexachloride[ALL] AND gamma[ALL]) OR (gamma[ALL] AND Benzene[ALL]) OR "Gamma 666"[ALL] OR Gammexane[ALL] OR "gamma HCH"[ALL] OR (gamma[ALL] AND Hexachlorocyclohexane[ALL]) OR "BHC Insecticide"[ALL] OR "Insecticide BHC"[ALL] OR "PMS Lindane"[ALL] OR (PMS[ALL] AND Lindane[ALL]) OR Kwell[ALL] OR Scabecid[ALL] OR Jacutin[ALL] OR Scabene[ALL] OR Tetocid[ALL] OR Scabisan[ALL] OR (Eta[ALL] AND hexachlorocyclohexane[ALL]) OR (Eta[ALL] AND hexachlorocyclohexane[ALL]) OR Delitex[ALL] OR acaricida[ALL] OR aparasin[ALL] OR aphthiria[ALL] OR aphtiria[ALL] OR atan[ALL] OR "battle bhc"[ALL] OR "benhex cream"[ALL] OR bhc[ALL] OR bicide[ALL] OR chloresene[ALL] OR dagicide[ALL] OR davesol[ALL] OR delice[ALL] OR delitex[ALL] OR devoran[ALL] OR elentol[ALL] OR entomoxan[ALL] OR forlin[ALL] OR gambex[ALL] OR gamene[ALL] OR gamiso[ALL] OR (gamma[ALL] AND hexachlorocyclohexane[ALL]) OR "gamma 666"[ALL] OR "gamma benzene"[ALL] OR "gamma bhc"[ALL] OR "gamma hch"[ALL] OR (gamma[ALL] AND hexachlorocyclohexane[ALL]) OR gammahexachlorcyclohexane[ALL] OR gammexane[ALL] OR geksan[ALL] OR gexane[ALL] OR hch[ALL] OR herklin[ALL] OR hexachloran[ALL] OR hexachlorane[ALL] OR hexaverm[ALL] OR hexchloran[ALL] OR hexicide[ALL] OR hexit[ALL] OR hexyclan[ALL] OR hisdane[ALL] OR jacuta[ALL] OR jacutin[ALL] OR kwell[ALL] OR "kwell lotion"[ALL] OR "kwell shampoo"[ALL] OR kwellada[ALL] OR lencid[ALL] OR lindactone[ALL] OR lindagam[ALL] OR linden[ALL] OR "linden lotion"[ALL] OR lorexane[ALL] OR (neo[ALL] AND scabicidol[ALL]) OR nourycid[ALL] OR "pms lindane"[ALL] OR quellada[ALL] OR (quellada[ALL] AND cream[ALL]) OR "quellada crème"[ALL] OR "quellada head"[ALL] OR "lice treatment"[ALL] OR "quellada lotion"[ALL] OR "quellada h"[ALL] OR sarconyl[ALL] OR scabecid[ALL] OR scabene[ALL] OR "scabene lotion"[ALL] OR scabexyl[ALL] OR scabi[ALL] OR scabien[ALL] OR scabisan[ALL] OR streunex[ALL] OR "tigal f"[ALL] OR "tri 6"[ALL] OR trisix[ALL] OR varsan[ALL] OR vermexane[ALL] OR veticide[ALL] OR methoxychlor[ALL] OR DMDT[ALL] OR (Dianisyl[ALL] AND Trichloroethane[ALL]) OR (Trichloroethane[ALL] AND Dianisyl[ALL]) OR Metox[ALL] OR (methoxyphenyl[ALL] AND ethane[ALL]) OR (methoxyphenyl[ALL] AND trichloroethane[ALL]) OR (methoxyphenyl[ALL] AND trichloroethane[ALL]) OR (anisyl[ALL] AND trichloroethane[ALL]) OR marlate[ALL] OR (methoxy[ALL] AND chlor[ALL]) OR (methoxy[ALL] AND ddt[ALL]) OR methoxyclor[ALL] OR mirex[ALL] OR (dodecachlorooctahydro[ALL] AND metheno[ALL]) OR (cyclobuta[ALL] AND pentalene[ALL]) OR mirax[ALL] OR perchloropentacyclodecane[ALL] OR nonachlor[ALL] OR (nonachloro[ALL] AND tetrahydro[ALL]) OR methanoindan[ALL] OR (nonachlor[ALL] AND isomer[ALL]) OR oxychlordane[ALL] OR (beta[ALL] AND octachloro[ALL]) OR (alpha[ALL] AND epoxy[ALL]) OR (alpha[ALL] AND tetrahydro[ALL]) OR oxychlordan[ALL] OR photomirex[ALL] OR monohydromirex[ALL] OR hydromirex[ALL] OR "mirex hydro"[ALL] OR (mirex[ALL] AND hydro[ALL]) OR "mirex monohydro"[ALL] OR (mirex[ALL] AND monohydro[ALL])) AND 1990/01/01:2023/09/01[dp]

EMBASE

(‘knockdown resistance’ OR (knockdown AND resistance) OR KDR) AND (‘Organochlorine insecticide’ OR (Organochlorine AND insecticide) OR ‘chlorinated insecticide’ OR ‘insecticide organochlorine’ OR ‘insecticides organochlorine’ OR (dichloro AND bis) OR (chlorophenyl AND ethane) OR (bis AND chlorophenyl) OR dichloroethane OR (para AND chlorophenyl) OR (bis AND dichloroethane) OR (dichloro AND ethane) OR (para AND ethylphenyl) OR (dichloroethylidene AND chlorobenzene) OR parachlorophenyl OR dichloroethane OR chlorophenyl OR dichloroethane OR ‘p chlorophenyl’ OR dichloroethane OR dichlloroethane OR dichlorethane OR dichloroethane OR ‘dichloroethane analog’ OR parachlorophenyl OR dichloroethane OR dichlorodiphenyldichloroethane OR DDD OR (dichlorodiphenyl AND dichloroethane) OR dichlorodiphenyldichloroethane OR dichlorodiphenyldichloroethane OR ddd OR (para AND ddd) OR tde OR dichloro OR (chlorophenyl AND ethylene) OR (chlorophenyl AND dichloroethylene) OR dichloroethylene OR (para AND chlorophenyl) OR ethylene OR (chlorophenyl AND ethylene) OR (dichloroethenylidene AND chlorobenzene) OR DDE OR (dichlorodiphenyl AND dichloroethylene) OR (para AND dde) OR trichloro OR chlorophenyl OR (chlorophenyl AND chlorophenyl) OR trichloroethane OR (ortho AND chlorophenyl) OR ‘op ddt’ OR ‘ortho ddt’ OR (ortho AND dichlorobenzene) OR dichlorobenzene OR Paradichlorobenzene OR ‘di chloricide’ OR dichloricide OR (para AND dichlorbenzene) OR (dichloro AND benzene) OR (para AND dichlorobenzene) OR paramoth OR Aldrin OR Isodrin OR (hexachloro AND hexahydro) OR dimethanonaphthalene OR (hexachloro AND hexahydro) OR aldrex OR ‘compound 118’ OR octalene OR photoaldrin OR (alpha AND hexachlorocyclohexane) OR ‘alpha HCH’ OR HCH OR (benzene AND hexachloride) OR (alpha AND benzenehexachlor) OR (alpha AND benzenehexachloride) OR ‘alpha bhc’ OR ‘alpha hch’ OR (beta AND hexachlorocyclohexane) OR cyclohexane OR (hexachloro AND beta) OR (beta AND hexachlorobenzene) OR (beta AND HCH) OR ‘beta Lindane’ OR (beta AND Lindane) OR ‘beta benzene’ OR (beta AND benzene) OR hexachloride OR ‘beta hexachloran’ OR (beta AND hexachloran) OR ‘beta 666’ OR ‘epsilon HCH’ OR ‘beta benzenehexachloride’ OR ‘beta bhc’ OR ‘beta hch’ OR campheclor OR alltox OR camfeclor OR camphechlor OR ‘camphene chlorinated’ OR ‘chlorinated camphene’ OR ‘chlorphen clorphen’ OR estonox OR geniphene OR penfene OR phenacide OR phenatox OR phenphene OR phenphere OR pinetox OR polychlorocamphene OR toxakil OR toxaphen OR toxaphene OR chlordane OR ‘gamma Chlordane’ OR Chlordane OR (octachloro AND alpha) OR (alpha AND hexahydro) OR methanoindene OR (octachloro AND alpha) OR (alpha AND tetrahydro) OR methanoindan OR (octachloro AND methane) OR (alpha AND tetrahydroindane) OR (alpha AND chlordane) OR alphachlordane OR ‘cd 68’ OR cd68 OR chlordane OR (cis AND chlordane) OR (cis AND photochlordane) OR ‘compound 1068’ OR ‘hcs 3260’ OR ‘m 410’ OR ‘octa klor’ OR (cis AND chlordane) OR photochlordane OR toxichlor OR (trans AND chlordane) OR ‘velsicol 1068’ OR wydane OR chlordecone OR Kepone OR decachlorooctahydro OR ‘metheno cyclobuta’ OR ‘pentalen one’ OR ‘cg 1189’ OR cg1189 OR kepone OR chlorphenotane OR chlorobenzene OR trichloroethylidene OR (chlorophenyl AND trichloroethane) OR trichloroethane OR trichloroethene OR benzochloryl OR chlorophenoltane OR chlorophenotane OR chlorophenothane OR chlorphenethanum OR chlorphenotan OR chlorphenothane OR chlorphenothanum OR clofenotan OR clofenotane OR d.d.t OR DDT OR ‘ddt residue’ OR detane OR dichlordiphenyltrichlormethylmethane OR dichlordiphenyltrichloroethane OR dichlorodiphenyltrichloroethane OR dicophane OR dodat OR esoderm OR estonate OR gesapon OR gesarex OR gesarol OR lentinol OR neocide OR ‘para ddt’ OR parachlorocide OR pentachlorin OR penticidum OR pestanal OR suleo OR ethane OR trichomon OR clofentezine OR bisclofentezin OR (chlorophenyl AND tetrazine) OR tetrazine OR dieldrin OR ‘Alvit 55’ OR Alvit55 OR (hexachloro AND epoxy) OR ‘octahydro endo’ OR ‘10 para’ OR ‘meta dieldrin’ OR ‘compound 497’ OR (dieldrin AND abavit) OR dieldrine OR heod OR photodieldrin OR endosulfan OR Thiodan OR Thiodon OR (beta AND Endosulfan) OR Thiotox OR (alpha AND Endosulfan) OR (hexachloronorbornene AND sulfite) OR (hydroxymethyl AND hexachloronorbornenesulfite) OR (hydroxymethyl AND hexachlorobicyclo) OR (ene AND sulfite) OR (hydroxymethyl AND hexachloronorcamphene) OR (alpha AND benzoepin) OR (alpha AND endosulfan) OR (alpha AND endosulfane) OR benzoepin OR beosit OR (beta AND benzoepin) OR (beta AND endosulfan) OR (beta AND endosulfane) OR chlorothiepine OR chlorthiapinum OR chlorthiepin OR cyclodan OR endogan OR ensawan OR ‘fmc 5462’ OR fmc5462 OR (hexachlorohexahydro AND methano) OR (benzodioxathiepine AND oxide) OR hexachloronorbornene OR (oxymethylene AND sulfite) OR malix OR sialan OR thifor OR thimul OR thiodan OR thionex OR thiotox OR thyodan OR thyonex OR tiodan OR endrin OR Hexadrin OR ‘compound 269’ OR (compound AND dieldrin) OR hexadrin OR heptachlor OR (heptachloro AND tetrahydro) OR methanoindene OR heptachlore OR heptachloro OR (tetrahydro AND methanoindene) OR photoheptachlor OR (heptachlor AND epoxide) OR (Epoxide AND Heptachlor) OR Heptachlorepoxide OR isobenzan OR (octachloro AND endomethylene) OR tetrahydrophthalan OR octachloro OR (hexahydro AND methanoisobenzofuran) OR (hexahydro AND methanoisobenzofuran) OR (octachloro AND hexahydro) OR methanoisobenzofuran OR (betahydro AND methanophthalan) OR izobenzan OR omtan OR ontan OR ‘r 6700’ OR ‘sd 4402’ OR telodrin OR lindane OR Hexachlorane OR (Benzene AND Hexachloride) OR (Hexachloride AND Benzene) OR (Epsilon AND hexachlorocyclohexane) OR (Epsilon AND hexachlorocyclohexane) OR (Zeta AND hexachlorocyclohexane) OR (Zeta AND hexachlorocyclohexane) OR Lindane OR (Benzene AND Hexachloride) OR (Hexachloride AND gamma) OR (gamma AND Benzene) OR ‘Gamma 666’ OR Gammexane OR ‘gamma HCH’ OR (gamma AND Hexachlorocyclohexane) OR ‘BHC Insecticide’ OR ‘Insecticide BHC’ OR ‘PMS Lindane’ OR (PMS AND Lindane) OR Kwell OR Scabecid OR Jacutin OR Scabene OR Tetocid OR Scabisan OR (Eta AND hexachlorocyclohexane) OR (Eta AND hexachlorocyclohexane) OR Delitex OR acaricida OR aparasin OR aphthiria OR aphtiria OR atan OR ‘battle bhc’ OR ‘benhex cream’ OR bhc OR bicide OR chloresene OR dagicide OR davesol OR delice OR delitex OR devoran OR elentol OR entomoxan OR forlin OR gambex OR gamene OR gamiso OR (gamma AND hexachlorocyclohexane) OR ‘gamma 666’ OR ‘gamma benzene’ OR ‘gamma bhc’ OR ‘gamma hch’ OR (gamma AND hexachlorocyclohexane) OR gammahexachlorcyclohexane OR gammexane OR geksan OR gexane OR hch OR herklin OR hexachloran OR hexachlorane OR hexaverm OR hexchloran OR hexicide OR hexit OR hexyclan OR hisdane OR jacuta OR jacutin OR kwell OR ‘kwell lotion’ OR ‘kwell shampoo’ OR kwellada OR lencid OR lindactone OR lindagam OR linden OR ‘linden lotion’ OR lorexane OR (neo AND scabicidol) OR nourycid OR ‘pms lindane’ OR quellada OR (quellada AND cream) OR ‘quellada crème’ OR ‘quellada head’ OR ‘lice treatment’ OR ‘quellada lotion’ OR ‘quellada h’ OR sarconyl OR scabecid OR scabene OR ‘scabene lotion’ OR scabexyl OR scabi OR scabien OR scabisan OR streunex OR ‘tigal f’ OR ‘tri 6’ OR trisix OR varsan OR vermexane OR veticide OR methoxychlor OR DMDT OR (Dianisyl AND Trichloroethane) OR (Trichloroethane AND Dianisyl) OR Metox OR (methoxyphenyl AND ethane) OR (methoxyphenyl AND trichloroethane) OR (methoxyphenyl AND trichloroethane) OR (anisyl AND trichloroethane) OR marlate OR (methoxy AND chlor) OR (methoxy AND ddt) OR methoxyclor OR mirex OR (dodecachlorooctahydro AND metheno) OR (cyclobuta AND pentalene) OR mirax OR perchloropentacyclodecane OR nonachlor OR (nonachloro AND tetrahydro) OR methanoindan OR (nonachlor AND isomer) OR oxychlordane OR (beta AND octachloro) OR (alpha AND epoxy) OR (alpha AND tetrahydro) OR oxychlordan OR photomirex OR monohydromirex OR hydromirex OR ‘mirex hydro’ OR (mirex AND hydro) OR ‘mirex monohydro’ OR (mirex AND monohydro)) AND [1990-2023]/PY

Scopus

(ALL(“knockdown resistance”) OR (ALL(knockdown) AND ALL(resistance))) OR KDR AND (ALL(Organochlorine) AND ALL(insecticide)) OR (ALL(Organochlorine) AND ALL(insecticide)) OR (ALL(chlorinated) AND ALL(insecticide)) OR ALL("insecticide organochlorine") OR ALL("insecticides organochlorine") OR (ALL(dichloro) AND ALL(bis)) OR (ALL(chlorophenyl) AND ALL(ethane)) OR (ALL(bis) AND ALL(chlorophenyl)) OR ALL(dichloroethane) OR (ALL(para) AND ALL(chlorophenyl)) OR (ALL(bis) AND ALL(dichloroethane)) OR (ALL(dichloro) AND ALL(ethane)) OR (ALL(para) AND ALL(ethylphenyl)) OR (ALL(dichloroethylidene) AND ALL(chlorobenzene)) OR ALL(parachlorophenyl) OR ALL(dichloroethane) OR ALL(chlorophenyl) OR ALL(dichloroethane) OR ALL("p chlorophenyl") OR ALL(dichloroethane) OR ALL(dichlloroethane) OR ALL(dichlorethane) OR ALL(dichloroethane) OR ALL("dichloroethane analog") OR ALL(parachlorophenyl) OR ALL(dichloroethane) OR ALL(dichlorodiphenyldichloroethane) OR ALL(DDD) OR (ALL(dichlorodiphenyl) AND ALL(dichloroethane)) OR ALL(dichlorodiphenyldichloroethane) OR ALL(dichlorodiphenyldichloroethane) OR ALL(ddd) OR (ALL(para) AND ALL(ddd)) OR ALL(tde) OR ALL(dichloro) OR (ALL(chlorophenyl) AND ALL(ethylene)) OR (ALL(chlorophenyl) AND ALL(dichloroethylene)) OR ALL(dichloroethylene) OR (ALL(para') AND ALL(chlorophenyl)) OR ALL(ethylene) OR (ALL(chlorophenyl) AND ALL(ethylene)) OR (ALL(dichloroethenylidene) AND ALL(chlorobenzene)) OR ALL(DDE) OR (ALL(dichlorodiphenyl) AND ALL(dichloroethylene)) OR (ALL(para) AND ALL(dde)) OR ALL(trichloro) OR ALL(chlorophenyl) OR (ALL(chlorophenyl) AND ALL(chlorophenyl)) OR ALL(trichloroethane) OR (ALL(ortho) AND ALL(chlorophenyl)) OR ALL("op ddt") OR ALL("ortho ddt") OR (ALL(ortho) AND ALL(dichlorobenzene)) OR ALL(dichlorobenzene) OR ALL(Paradichlorobenzene) OR ALL("di chloricide") OR ALL(dichloricide) OR (ALL(para) AND ALL(dichlorbenzene)) OR (ALL(dichloro) AND ALL(benzene)) OR (ALL(para) AND ALL(dichlorobenzene)) OR ALL(paramoth) OR ALL(Aldrin) OR ALL(Isodrin) OR (ALL(hexachloro) AND ALL(hexahydro)) OR ALL(dimethanonaphthalene) OR (ALL(hexachloro) AND ALL(hexahydro)) OR ALL(aldrex) OR ALL("compound 118") OR ALL(octalene) OR ALL(photoaldrin) OR (ALL(alpha) AND ALL(hexachlorocyclohexane)) OR ALL("alpha HCH") OR ALL(HCH) OR (ALL(benzene) AND ALL(hexachloride)) OR (ALL(alpha) AND ALL(benzenehexachlor)) OR (ALL(alpha) AND ALL(benzenehexachloride)) OR ALL("alpha bhc") OR ALL("alpha hch") OR (ALL(beta) AND ALL(hexachlorocyclohexane)) OR ALL(cyclohexane) OR (ALL(hexachloro) AND ALL(beta)) OR (ALL(beta) AND ALL(hexachlorobenzene)) OR (ALL(beta) AND ALL(HCH)) OR ALL("beta Lindane") OR (ALL(beta) AND ALL(Lindane)) OR ALL("beta benzene") OR (ALL(beta) AND ALL(benzene)) OR ALL(hexachloride) OR ALL("beta hexachloran") OR (ALL(beta) AND ALL(hexachloran)) OR ALL("beta 666") OR ALL("epsilon HCH") OR (ALL(beta) AND ALL(benzenehexachloride)) OR ALL("beta bhc") OR ALL("beta hch") OR ALL(campheclor) OR ALL(alltox) OR ALL(camfeclor) OR ALL(camphechlor) OR (ALL(camphene) AND ALL(chlorinated)) OR ALL("chlorinated camphene") OR ALL("chlorphen clorphen") OR ALL(estonox) OR ALL(geniphene) OR ALL(penfene) OR ALL(phenacide) OR ALL(phenatox) OR ALL(phenphene) OR ALL(phenphere) OR ALL(pinetox) OR ALL(polychlorocamphene) OR ALL(toxakil) OR ALL(toxaphen) OR ALL(toxaphene) OR ALL(chlordane) OR ALL("gamma Chlordane") OR ALL(Chlordane) OR (ALL(octachloro) AND ALL(alpha)) OR (ALL(alpha) AND ALL(hexahydro)) OR ALL(methanoindene) OR (ALL(octachloro) AND ALL(alpha)) OR (ALL(alpha) AND ALL(tetrahydro)) OR ALL(methanoindan) OR (ALL(octachloro) AND ALL(methane)) OR (ALL(alpha) AND ALL(tetrahydroindane)) OR (ALL(alpha) AND ALL(chlordane)) OR ALL(alphachlordane) OR ALL("cd 68") OR ALL(cd68) OR ALL(chlordane) OR (ALL(cis) AND ALL(chlordane)) OR (ALL(cis) AND ALL(photochlordane)) OR ALL("compound 1068") OR ALL("hcs 3260") OR ALL("m 410") OR ALL("octa klor") OR (ALL(cis) AND ALL(chlordane)) OR ALL(photochlordane) OR ALL(toxichlor) OR (ALL(trans) AND ALL(chlordane)) OR ALL("velsicol 1068") OR ALL(wydane) OR ALL(chlordecone) OR ALL(Kepone) OR ALL(decachlorooctahydro) OR ALL("metheno cyclobuta") OR ALL("pentalen one") OR ALL("cg 1189") OR ALL(cg1189) OR ALL(kepone) OR ALL(chlorphenotane) OR ALL(chlorobenzene) OR ALL(trichloroethylidene) OR (ALL(chlorophenyl) AND ALL(trichloroethane)) OR ALL(trichloroethane) OR ALL(trichloroethene) OR ALL(benzochloryl) OR ALL(chlorophenoltane) OR ALL(chlorophenotane) OR ALL(chlorophenothane) OR ALL(chlorphenethanum) OR ALL(chlorphenotan) OR ALL(chlorphenothane) OR ALL(chlorphenothanum) OR ALL(clofenotan) OR ALL(clofenotane) OR ALL(d.d.t) OR ALL(DDT) OR ALL("ddt residue") OR ALL(detane) OR ALL(dichlordiphenyltrichlormethylmethane) OR ALL(dichlordiphenyltrichloroethane) OR ALL(dichlorodiphenyltrichloroethane) OR ALL(dicophane) OR ALL(dodat) OR ALL(esoderm) OR ALL(estonate) OR ALL(gesapon) OR ALL(gesarex) OR ALL(gesarol) OR ALL(lentinol) OR ALL(neocide) OR ALL("para ddt") OR ALL(parachlorocide) OR ALL(pentachlorin) OR ALL(penticidum) OR ALL(pestanal) OR ALL(suleo) OR ALL(ethane) OR ALL(trichomon) OR ALL(clofentezine) OR ALL(bisclofentezin) OR (ALL(chlorophenyl) AND ALL(tetrazine)) OR ALL(tetrazine) OR ALL(dieldrin) OR ALL("Alvit 55") OR ALL(Alvit55) OR (ALL(hexachloro) AND ALL(epoxy)) OR ALL("octahydro endo") OR ALL("10 para") OR ALL("meta dieldrin") OR ALL("compound 497") OR (ALL(dieldrin) AND ALL(abavit)) OR ALL(dieldrine) OR ALL(heod) OR ALL(photodieldrin) OR ALL(endosulfan) OR ALL(Thiodan) OR ALL(Thiodon) OR (ALL(beta) AND ALL(Endosulfan)) OR ALL(Thiotox) OR (ALL(alpha) AND ALL(Endosulfan)) OR (ALL(hexachloronorbornene) AND ALL(sulfite)) OR (ALL(hydroxymethyl) AND ALL(hexachloronorbornenesulfite)) OR (ALL(hydroxymethyl) AND ALL(hexachlorobicyclo)) OR (ALL(ene) AND ALL(sulfite)) OR (ALL(hydroxymethyl) AND ALL(hexachloronorcamphene)) OR (ALL(alpha) AND ALL(benzoepin)) OR (ALL(alpha) AND ALL(endosulfan)) OR (ALL(alpha) AND ALL(endosulfane)) OR ALL(benzoepin) OR ALL(beosit) OR (ALL(beta) AND ALL(benzoepin)) OR (ALL(beta) AND ALL(endosulfan)) OR (ALL(beta) AND ALL(endosulfane)) OR ALL(chlorothiepine) OR ALL(chlorthiapinum) OR ALL(chlorthiepin) OR ALL(cyclodan) OR ALL(endogan) OR ALL(ensawan) OR ALL("fmc 5462") OR ALL(fmc5462) OR (ALL(hexachlorohexahydro) AND ALL(methano)) OR (ALL(benzodioxathiepine) AND ALL(oxide)) OR ALL(hexachloronorbornene) OR (ALL(oxymethylene) AND ALL(sulfite)) OR ALL(malix) OR ALL(sialan) OR ALL(thifor) OR ALL(thimul) OR ALL(thiodan) OR ALL(thionex) OR ALL(thiotox) OR ALL(thyodan) OR ALL(thyonex) OR ALL(tiodan) OR ALL(endrin) OR ALL(Hexadrin) OR ALL("compound 269") OR (ALL(compound) AND ALL(dieldrin)) OR ALL(hexadrin) OR ALL(heptachlor) OR (ALL(heptachloro) AND ALL(tetrahydro)) OR ALL(methanoindene) OR ALL(heptachlore) OR ALL(heptachloro) OR (ALL(tetrahydro) AND ALL(methanoindene)) OR ALL(photoheptachlor) OR (ALL(heptachlor) AND ALL(epoxide)) OR (ALL(Epoxide) AND ALL(Heptachlor)) OR ALL(Heptachlorepoxide) OR ALL(isobenzan) OR (ALL(octachloro) AND ALL(endomethylene)) OR ALL(tetrahydrophthalan) OR ALL(octachloro) OR (ALL(hexahydro) AND ALL(methanoisobenzofuran)) OR (ALL(hexahydro) AND ALL(methanoisobenzofuran)) OR (ALL(octachloro) AND ALL(hexahydro)) OR ALL(methanoisobenzofuran) OR (ALL(betahydro) AND ALL(methanophthalan)) OR ALL(izobenzan) OR ALL(omtan) OR ALL(ontan) OR ALL("r 6700") OR ALL("sd 4402") OR ALL(telodrin) OR ALL(lindane) OR ALL(Hexachlorane) OR (ALL(Benzene) AND ALL(Hexachloride)) OR (ALL(Hexachloride) AND ALL(Benzene)) OR (ALL(Epsilon) AND ALL(hexachlorocyclohexane)) OR (ALL(Epsilon) AND ALL(hexachlorocyclohexane)) OR (ALL(Zeta) AND ALL(hexachlorocyclohexane)) OR (ALL(Zeta) AND ALL(hexachlorocyclohexane)) OR ALL(Lindane) OR (ALL(Benzene) AND ALL(Hexachloride)) OR (ALL(Hexachloride) AND ALL(gamma)) OR (ALL(gamma) AND ALL(Benzene)) OR ALL("Gamma 666") OR ALL(Gammexane) OR ALL("gamma HCH") OR (ALL(gamma) AND ALL(Hexachlorocyclohexane)) OR ALL("BHC Insecticide") OR ALL("Insecticide BHC") OR ALL("PMS Lindane") OR (ALL(PMS) AND ALL(Lindane)) OR ALL(Kwell) OR ALL(Scabecid) OR ALL(Jacutin) OR ALL(Scabene) OR ALL(Tetocid) OR ALL(Scabisan) OR (ALL(Eta) AND ALL(hexachlorocyclohexane)) OR (ALL(Eta) AND ALL(hexachlorocyclohexane)) OR ALL(Delitex) OR ALL(acaricida) OR ALL(aparasin) OR ALL(aphthiria) OR ALL(aphtiria) OR ALL(atan) OR ALL("battle bhc") OR ALL("benhex cream") OR ALL(bhc) OR ALL(bicide) OR ALL(chloresene) OR ALL(dagicide) OR ALL(davesol) OR ALL(delice) OR ALL(delitex) OR ALL(devoran) OR ALL(elentol) OR ALL(entomoxan) OR ALL(forlin) OR ALL(gambex) OR ALL(gamene) OR ALL(gamiso) OR (ALL(gamma) AND ALL(hexachlorocyclohexane)) OR ALL("gamma 666") OR ALL("gamma benzene") OR ALL("gamma bhc") OR ALL("gamma hch") OR (ALL(gamma) AND ALL(hexachlorocyclohexane)) OR ALL(gammahexachlorcyclohexane) OR ALL(gammexane) OR ALL(geksan) OR ALL(gexane) OR ALL(hch) OR ALL(herklin) OR ALL(hexachloran) OR ALL(hexachlorane) OR ALL(hexaverm) OR ALL(hexchloran) OR ALL(hexicide) OR ALL(hexit) OR ALL(hexyclan) OR ALL(hisdane) OR ALL(jacuta) OR ALL(jacutin) OR ALL(kwell) OR ALL("kwell lotion") OR ALL("kwell shampoo") OR ALL(kwellada) OR ALL(lencid) OR ALL(lindactone) OR ALL(lindagam) OR ALL(linden) OR ALL("linden lotion") OR ALL(lorexane) OR (ALL(neo) AND ALL(scabicidol)) OR ALL(nourycid) OR ALL("pms lindane") OR ALL(quellada) OR (ALL(quellada) AND ALL(cream)) OR ALL("quellada crème") OR ALL("quellada head") OR ALL("lice treatment") OR ALL("quellada lotion") OR ALL("quellada h") OR ALL(sarconyl) OR ALL(scabecid) OR ALL(scabene) OR ALL("scabene lotion") OR ALL(scabexyl) OR ALL(scabi) OR ALL(scabien) OR ALL(scabisan) OR ALL(streunex) OR ALL("tigal f") OR ALL("tri 6") OR ALL(trisix) OR ALL(varsan) OR ALL(vermexane) OR ALL(veticide) OR ALL(methoxychlor) OR ALL(DMDT) OR (ALL(Dianisyl) AND ALL(Trichloroethane)) OR (ALL(Trichloroethane) AND ALL(Dianisyl)) OR ALL(Metox) OR (ALL(methoxyphenyl) AND ALL(ethane)) OR (ALL(methoxyphenyl) AND ALL(trichloroethane)) OR (ALL(methoxyphenyl) AND ALL(trichloroethane)) OR (ALL(anisyl) AND ALL(trichloroethane)) OR ALL(marlate) OR (ALL(methoxy) AND ALL(chlor)) OR (ALL(methoxy) AND ALL(ddt)) OR ALL(methoxyclor) OR ALL(mirex) OR (ALL(dodecachlorooctahydro) AND ALL(metheno)) OR (ALL(cyclobuta) AND ALL(pentalene)) OR ALL(mirax) OR ALL(perchloropentacyclodecane) OR ALL(nonachlor) OR (ALL(nonachloro) AND ALL(tetrahydro)) OR ALL(methanoindan) OR (ALL(nonachlor) AND ALL(isomer)) OR ALL(oxychlordane) OR (ALL(beta) AND ALL(octachloro)) OR (ALL(alpha) AND ALL(epoxy)) OR (ALL(alpha) AND ALL(tetrahydro)) OR ALL(oxychlordan) OR ALL(photomirex) OR ALL(monohydromirex) OR ALL(hydromirex) OR ALL("mirex hydro") OR (ALL(mirex) AND ALL(hydro)) OR ALL("mirex monohydro") OR (ALL(mirex) AND ALL(monohydro)) AND PUBYEAR < 2024 AND PUBYEAR > 1989 AND NOT PUBDATETXT ("October 2020" OR "November 2020" OR "December 2020")

Wos

(ALL=(“knockdown resistance”) OR (ALL=(knockdown) AND ALL=(resistance)) OR ALL=(KDR)) AND (ALL=(Organochlorine) AND ALL=(insecticide)) OR (ALL=(Organochlorine) AND ALL=(insecticide)) OR (ALL=(chlorinated) AND ALL=(insecticide)) OR ALL=("insecticide organochlorine") OR ALL=("insecticides organochlorine") OR (ALL=(dichloro) AND ALL=(bis)) OR (ALL=(chlorophenyl) AND ALL=(ethane)) OR (ALL=(bis) AND ALL=(chlorophenyl)) OR ALL=(dichloroethane) OR (ALL=(para) AND ALL=(chlorophenyl)) OR (ALL=(bis) AND ALL=(dichloroethane)) OR (ALL=(dichloro) AND ALL=(ethane)) OR (ALL=(para) AND ALL=(ethylphenyl)) OR (ALL=(dichloroethylidene) AND ALL=(chlorobenzene)) OR ALL=(parachlorophenyl) OR ALL=(dichloroethane) OR ALL=(chlorophenyl) OR ALL=(dichloroethane) OR ALL=("p chlorophenyl") OR ALL=(dichloroethane) OR ALL=(dichlloroethane) OR ALL=(dichlorethane) OR ALL=(dichloroethane) OR ALL=("dichloroethane analog") OR ALL=(parachlorophenyl) OR ALL=(dichloroethane) OR ALL=(dichlorodiphenyldichloroethane) OR ALL=(DDD) OR (ALL=(dichlorodiphenyl) AND ALL=(dichloroethane)) OR ALL=(dichlorodiphenyldichloroethane) OR ALL=(dichlorodiphenyldichloroethane) OR ALL=(ddd) OR (ALL=(para) AND ALL=(ddd)) OR ALL=(tde) OR ALL=(dichloro) OR (ALL=(chlorophenyl) AND ALL=(ethylene)) OR (ALL=(chlorophenyl) AND ALL=(dichloroethylene)) OR ALL=(dichloroethylene) OR (ALL=(para') AND ALL=(chlorophenyl)) OR ALL=(ethylene) OR (ALL=(chlorophenyl) AND ALL=(ethylene)) OR (ALL=(dichloroethenylidene) AND ALL=(chlorobenzene)) OR ALL=(DDE) OR (ALL=(dichlorodiphenyl) AND ALL=(dichloroethylene)) OR (ALL=(para) AND ALL=(dde)) OR ALL=(trichloro) OR ALL=(chlorophenyl) OR (ALL=(chlorophenyl) AND ALL=(chlorophenyl)) OR ALL=(trichloroethane) OR (ALL=(ortho) AND ALL=(chlorophenyl)) OR ALL=("op ddt") OR ALL=("ortho ddt") OR (ALL=(ortho) AND ALL=(dichlorobenzene)) OR ALL=(dichlorobenzene) OR ALL=(Paradichlorobenzene) OR ALL=("di chloricide") OR ALL=(dichloricide) OR (ALL=(para) AND ALL=(dichlorbenzene)) OR (ALL=(dichloro) AND ALL=(benzene)) OR (ALL=(para) AND ALL=(dichlorobenzene)) OR ALL=(paramoth) OR ALL=(Aldrin) OR ALL=(Isodrin) OR (ALL=(hexachloro) AND ALL=(hexahydro)) OR ALL=(dimethanonaphthalene) OR (ALL=(hexachloro) AND ALL=(hexahydro)) OR ALL=(aldrex) OR ALL=("compound 118") OR ALL=(octalene) OR ALL=(photoaldrin) OR (ALL=(alpha) AND ALL=(hexachlorocyclohexane)) OR ALL=("alpha HCH") OR ALL=(HCH) OR (ALL=(benzene) AND ALL=(hexachloride)) OR (ALL=(alpha) AND ALL=(benzenehexachlor)) OR (ALL=(alpha) AND ALL=(benzenehexachloride)) OR ALL=("alpha bhc") OR ALL=("alpha hch") OR (ALL=(beta) AND ALL=(hexachlorocyclohexane)) OR ALL=(cyclohexane) OR (ALL=(hexachloro) AND ALL=(beta)) OR (ALL=(beta) AND ALL=(hexachlorobenzene)) OR (ALL=(beta) AND ALL=(HCH)) OR ALL=("beta Lindane") OR (ALL=(beta) AND ALL=(Lindane)) OR ALL=("beta benzene") OR (ALL=(beta) AND ALL=(benzene)) OR ALL=(hexachloride) OR ALL=("beta hexachloran") OR (ALL=(beta) AND ALL=(hexachloran)) OR ALL=("beta 666") OR ALL=("epsilon HCH") OR (ALL=(beta) AND ALL=(benzenehexachloride)) OR ALL=("beta bhc") OR ALL=("beta hch") OR ALL=(campheclor) OR ALL=(alltox) OR ALL=(camfeclor) OR ALL=(camphechlor) OR (ALL=(camphene) AND ALL=(chlorinated)) OR ALL=("chlorinated camphene") OR ALL=("chlorphen clorphen") OR ALL=(estonox) OR ALL=(geniphene) OR ALL=(penfene) OR ALL=(phenacide) OR ALL=(phenatox) OR ALL=(phenphene) OR ALL=(phenphere) OR ALL=(pinetox) OR ALL=(polychlorocamphene) OR ALL=(toxakil) OR ALL=(toxaphen) OR ALL=(toxaphene) OR ALL=(chlordane) OR ALL=("gamma Chlordane") OR ALL=(Chlordane) OR (ALL=(octachloro) AND ALL=(alpha)) OR (ALL=(alpha) AND ALL=(hexahydro)) OR ALL=(methanoindene) OR (ALL=(octachloro) AND ALL=(alpha)) OR (ALL=(alpha) AND ALL=(tetrahydro)) OR ALL=(methanoindan) OR (ALL=(octachloro) AND ALL=(methane)) OR (ALL=(alpha) AND ALL=(tetrahydroindane)) OR (ALL=(alpha) AND ALL=(chlordane)) OR ALL=(alphachlordane) OR ALL=("cd 68") OR ALL=(cd68) OR ALL=(chlordane) OR (ALL=(cis) AND ALL=(chlordane)) OR (ALL=(cis) AND ALL=(photochlordane)) OR ALL=("compound 1068") OR ALL=("hcs 3260") OR ALL=("m 410") OR ALL=("octa klor") OR (ALL=(cis) AND ALL=(chlordane)) OR ALL=(photochlordane) OR ALL=(toxichlor) OR (ALL=(trans) AND ALL=(chlordane)) OR ALL=("velsicol 1068") OR ALL=(wydane) OR ALL=(chlordecone) OR ALL=(Kepone) OR ALL=(decachlorooctahydro) OR ALL=("metheno cyclobuta") OR ALL=("pentalen one") OR ALL=("cg 1189") OR ALL=(cg1189) OR ALL=(kepone) OR ALL=(chlorphenotane) OR ALL=(chlorobenzene) OR ALL=(trichloroethylidene) OR (ALL=(chlorophenyl) AND ALL=(trichloroethane)) OR ALL=(trichloroethane) OR ALL=(trichloroethene) OR ALL=(benzochloryl) OR ALL=(chlorophenoltane) OR ALL=(chlorophenotane) OR ALL=(chlorophenothane) OR ALL=(chlorphenethanum) OR ALL=(chlorphenotan) OR ALL=(chlorphenothane) OR ALL=(chlorphenothanum) OR ALL=(clofenotan) OR ALL=(clofenotane) OR ALL=(d.d.t) OR ALL=(DDT) OR ALL=("ddt residue") OR ALL=(detane) OR ALL=(dichlordiphenyltrichlormethylmethane) OR ALL=(dichlordiphenyltrichloroethane) OR ALL=(dichlorodiphenyltrichloroethane) OR ALL=(dicophane) OR ALL=(dodat) OR ALL=(esoderm) OR ALL=(estonate) OR ALL=(gesapon) OR ALL=(gesarex) OR ALL=(gesarol) OR ALL=(lentinol) OR ALL=(neocide) OR ALL=("para ddt") OR ALL=(parachlorocide) OR ALL=(pentachlorin) OR ALL=(penticidum) OR ALL=(pestanal) OR ALL=(suleo) OR ALL=(ethane) OR ALL=(trichomon) OR ALL=(clofentezine) OR ALL=(bisclofentezin) OR (ALL=(chlorophenyl) AND ALL=(tetrazine)) OR ALL=(tetrazine) OR ALL=(dieldrin) OR ALL=("Alvit 55") OR ALL=(Alvit55) OR (ALL=(hexachloro) AND ALL=(epoxy)) OR ALL=("octahydro endo") OR ALL=("10 para") OR ALL=("meta dieldrin") OR ALL=("compound 497") OR (ALL=(dieldrin) AND ALL=(abavit)) OR ALL=(dieldrine) OR ALL=(heod) OR ALL=(photodieldrin) OR ALL=(endosulfan) OR ALL=(Thiodan) OR ALL=(Thiodon) OR (ALL=(beta) AND ALL=(Endosulfan)) OR ALL=(Thiotox) OR (ALL=(alpha) AND ALL=(Endosulfan)) OR (ALL=(hexachloronorbornene) AND ALL=(sulfite)) OR (ALL=(hydroxymethyl) AND ALL=(hexachloronorbornenesulfite)) OR (ALL=(hydroxymethyl) AND ALL=(hexachlorobicyclo)) OR (ALL=(ene) AND ALL=(sulfite)) OR (ALL=(hydroxymethyl) AND ALL=(hexachloronorcamphene)) OR (ALL=(alpha) AND ALL=(benzoepin)) OR (ALL=(alpha) AND ALL=(endosulfan)) OR (ALL=(alpha) AND ALL=(endosulfane)) OR ALL=(benzoepin) OR ALL=(beosit) OR (ALL=(beta) AND ALL=(benzoepin)) OR (ALL=(beta) AND ALL=(endosulfan)) OR (ALL=(beta) AND ALL=(endosulfane)) OR ALL=(chlorothiepine) OR ALL=(chlorthiapinum) OR ALL=(chlorthiepin) OR ALL=(cyclodan) OR ALL=(endogan) OR ALL=(ensawan) OR ALL=("fmc 5462") OR ALL=(fmc5462) OR (ALL=(hexachlorohexahydro) AND ALL=(methano)) OR (ALL=(benzodioxathiepine) AND ALL=(oxide)) OR ALL=(hexachloronorbornene) OR (ALL=(oxymethylene) AND ALL=(sulfite)) OR ALL=(malix) OR ALL=(sialan) OR ALL=(thifor) OR ALL=(thimul) OR ALL=(thiodan) OR ALL=(thionex) OR ALL=(thiotox) OR ALL=(thyodan) OR ALL=(thyonex) OR ALL=(tiodan) OR ALL=(endrin) OR ALL=(Hexadrin) OR ALL=("compound 269") OR (ALL=(compound) AND ALL=(dieldrin)) OR ALL=(hexadrin) OR ALL=(heptachlor) OR (ALL=(heptachloro) AND ALL=(tetrahydro)) OR ALL=(methanoindene) OR ALL=(heptachlore) OR ALL=(heptachloro) OR (ALL=(tetrahydro) AND ALL=(methanoindene)) OR ALL=(photoheptachlor) OR (ALL=(heptachlor) AND ALL=(epoxide)) OR (ALL=(Epoxide) AND ALL=(Heptachlor)) OR ALL=(Heptachlorepoxide) OR ALL=(isobenzan) OR (ALL=(octachloro) AND ALL=(endomethylene)) OR ALL=(tetrahydrophthalan) OR ALL=(octachloro) OR (ALL=(hexahydro) AND ALL=(methanoisobenzofuran)) OR (ALL=(hexahydro) AND ALL=(methanoisobenzofuran)) OR (ALL=(octachloro) AND ALL=(hexahydro)) OR ALL=(methanoisobenzofuran) OR (ALL=(betahydro) AND ALL=(methanophthalan)) OR ALL=(izobenzan) OR ALL=(omtan) OR ALL=(ontan) OR ALL=("r 6700") OR ALL=("sd 4402") OR ALL=(telodrin) OR ALL=(lindane) OR ALL=(Hexachlorane) OR (ALL=(Benzene) AND ALL=(Hexachloride)) OR (ALL=(Hexachloride) AND ALL=(Benzene)) OR (ALL=(Epsilon) AND ALL=(hexachlorocyclohexane)) OR (ALL=(Epsilon) AND ALL=(hexachlorocyclohexane)) OR (ALL=(Zeta) AND ALL=(hexachlorocyclohexane)) OR (ALL=(Zeta) AND ALL=(hexachlorocyclohexane)) OR ALL=(Lindane) OR (ALL=(Benzene) AND ALL=(Hexachloride)) OR (ALL=(Hexachloride) AND ALL=(gamma)) OR (ALL=(gamma) AND ALL=(Benzene)) OR ALL=("Gamma 666") OR ALL=(Gammexane) OR ALL=("gamma HCH") OR (ALL=(gamma) AND ALL=(Hexachlorocyclohexane)) OR ALL=("BHC Insecticide") OR ALL=("Insecticide BHC") OR ALL=("PMS Lindane") OR (ALL=(PMS) AND ALL=(Lindane)) OR ALL=(Kwell) OR ALL=(Scabecid) OR ALL=(Jacutin) OR ALL=(Scabene) OR ALL=(Tetocid) OR ALL=(Scabisan) OR (ALL=(Eta) AND ALL=(hexachlorocyclohexane)) OR (ALL=(Eta) AND ALL=(hexachlorocyclohexane)) OR ALL=(Delitex) OR ALL=(acaricida) OR ALL=(aparasin) OR ALL=(aphthiria) OR ALL=(aphtiria) OR ALL=(atan) OR ALL=("battle bhc") OR ALL=("benhex cream") OR ALL=(bhc) OR ALL=(bicide) OR ALL=(chloresene) OR ALL=(dagicide) OR ALL=(davesol) OR ALL=(delice) OR ALL=(delitex) OR ALL=(devoran) OR ALL=(elentol) OR ALL=(entomoxan) OR ALL=(forlin) OR ALL=(gambex) OR ALL=(gamene) OR ALL=(gamiso) OR (ALL=(gamma) AND ALL=(hexachlorocyclohexane)) OR ALL=("gamma 666") OR ALL=("gamma benzene") OR ALL=("gamma bhc") OR ALL=("gamma hch") OR (ALL=(gamma) AND ALL=(hexachlorocyclohexane)) OR ALL=(gammahexachlorcyclohexane) OR ALL=(gammexane) OR ALL=(geksan) OR ALL=(gexane) OR ALL=(hch) OR ALL=(herklin) OR ALL=(hexachloran) OR ALL=(hexachlorane) OR ALL=(hexaverm) OR ALL=(hexchloran) OR ALL=(hexicide) OR ALL=(hexit) OR ALL=(hexyclan) OR ALL=(hisdane) OR ALL=(jacuta) OR ALL=(jacutin) OR ALL=(kwell) OR ALL=("kwell lotion") OR ALL=("kwell shampoo") OR ALL=(kwellada) OR ALL=(lencid) OR ALL=(lindactone) OR ALL=(lindagam) OR ALL=(linden) OR ALL=("linden lotion") OR ALL=(lorexane) OR (ALL=(neo) AND ALL=(scabicidol)) OR ALL=(nourycid) OR ALL=("pms lindane") OR ALL=(quellada) OR (ALL=(quellada) AND ALL=(cream)) OR ALL=("quellada crème") OR ALL=("quellada head") OR ALL=("lice treatment") OR ALL=("quellada lotion") OR ALL=("quellada h") OR ALL=(sarconyl) OR ALL=(scabecid) OR ALL=(scabene) OR ALL=("scabene lotion") OR ALL=(scabexyl) OR ALL=(scabi) OR ALL=(scabien) OR ALL=(scabisan) OR ALL=(streunex) OR ALL=("tigal f") OR ALL=("tri 6") OR ALL=(trisix) OR ALL=(varsan) OR ALL=(vermexane) OR ALL=(veticide) OR ALL=(methoxychlor) OR ALL=(DMDT) OR (ALL=(Dianisyl) AND ALL=(Trichloroethane)) OR (ALL=(Trichloroethane) AND ALL=(Dianisyl)) OR ALL=(Metox) OR (ALL=(methoxyphenyl) AND ALL=(ethane)) OR (ALL=(methoxyphenyl) AND ALL=(trichloroethane)) OR (ALL=(methoxyphenyl) AND ALL=(trichloroethane)) OR (ALL=(anisyl) AND ALL=(trichloroethane)) OR ALL=(marlate) OR (ALL=(methoxy) AND ALL=(chlor)) OR (ALL=(methoxy) AND ALL=(ddt)) OR ALL=(methoxyclor) OR ALL=(mirex) OR (ALL=(dodecachlorooctahydro) AND ALL=(metheno)) OR (ALL=(cyclobuta) AND ALL=(pentalene)) OR ALL=(mirax) OR ALL=(perchloropentacyclodecane) OR ALL=(nonachlor) OR (ALL=(nonachloro) AND ALL=(tetrahydro)) OR ALL=(methanoindan) OR (ALL=(nonachlor) AND ALL=(isomer)) OR ALL=(oxychlordane) OR (ALL=(beta) AND ALL=(octachloro)) OR (ALL=(alpha) AND ALL=(epoxy)) OR (ALL=(alpha) AND ALL=(tetrahydro)) OR ALL=(oxychlordan) OR ALL=(photomirex) OR ALL=(monohydromirex) OR ALL=(hydromirex) OR ALL=("mirex hydro") OR (ALL=(mirex) AND ALL=(hydro)) OR ALL=("mirex monohydro") OR (ALL=(mirex) AND ALL=(monohydro)) AND PY=(1990-2023)

Proquest

ALL,FT(“knockdown resistance”) OR (ALL,FT(knockdown) AND ALL,FT(resistance)) OR ALL,FT(KDR) AND (ALL,FT(Organochlorine) AND ALL,FT(insecticide)) OR (ALL,FT(Organochlorine) AND ALL,FT(insecticide)) OR (ALL,FT(chlorinated) AND ALL,FT(insecticide)) OR ALL,FT("insecticide organochlorine") OR ALL,FT("insecticides organochlorine") OR (ALL,FT(dichloro) AND ALL,FT(bis)) OR (ALL,FT(chlorophenyl) AND ALL,FT(ethane)) OR (ALL,FT(bis) AND ALL,FT(chlorophenyl)) OR ALL,FT(dichloroethane) OR (ALL,FT(para) AND ALL,FT(chlorophenyl)) OR (ALL,FT(bis) AND ALL,FT(dichloroethane)) OR (ALL,FT(dichloro) AND ALL,FT(ethane)) OR (ALL,FT(para) AND ALL,FT(ethylphenyl)) OR (ALL,FT(dichloroethylidene) AND ALL,FT(chlorobenzene)) OR ALL,FT(parachlorophenyl) OR ALL,FT(dichloroethane) OR ALL,FT(chlorophenyl) OR ALL,FT(dichloroethane) OR ALL,FT("p chlorophenyl") OR ALL,FT(dichloroethane) OR ALL,FT(dichlloroethane) OR ALL,FT(dichlorethane) OR ALL,FT(dichloroethane) OR ALL,FT("dichloroethane analog") OR ALL,FT(parachlorophenyl) OR ALL,FT(dichloroethane) OR ALL,FT(dichlorodiphenyldichloroethane) OR ALL,FT(DDD) OR (ALL,FT(dichlorodiphenyl) AND ALL,FT(dichloroethane)) OR ALL,FT(dichlorodiphenyldichloroethane) OR ALL,FT(dichlorodiphenyldichloroethane) OR ALL,FT(ddd) OR (ALL,FT(para) AND ALL,FT(ddd)) OR ALL,FT(tde) OR ALL,FT(dichloro) OR (ALL,FT(chlorophenyl) AND ALL,FT(ethylene)) OR (ALL,FT(chlorophenyl) AND ALL,FT(dichloroethylene)) OR ALL,FT(dichloroethylene) OR (ALL,FT(para') AND ALL,FT(chlorophenyl)) OR ALL,FT(ethylene) OR (ALL,FT(chlorophenyl) AND ALL,FT(ethylene)) OR (ALL,FT(dichloroethenylidene) AND ALL,FT(chlorobenzene)) OR ALL,FT(DDE) OR (ALL,FT(dichlorodiphenyl) AND ALL,FT(dichloroethylene)) OR (ALL,FT(para) AND ALL,FT(dde)) OR ALL,FT(trichloro) OR ALL,FT(chlorophenyl) OR (ALL,FT(chlorophenyl) AND ALL,FT(chlorophenyl)) OR ALL,FT(trichloroethane) OR (ALL,FT(ortho) AND ALL,FT(chlorophenyl)) OR ALL,FT("op ddt") OR ALL,FT("ortho ddt") OR (ALL,FT(ortho) AND ALL,FT(dichlorobenzene)) OR ALL,FT(dichlorobenzene) OR ALL,FT(Paradichlorobenzene) OR ALL,FT("di chloricide") OR ALL,FT(dichloricide) OR (ALL,FT(para) AND ALL,FT(dichlorbenzene)) OR (ALL,FT(dichloro) AND ALL,FT(benzene)) OR (ALL,FT(para) AND ALL,FT(dichlorobenzene)) OR ALL,FT(paramoth) OR ALL,FT(Aldrin) OR ALL,FT(Isodrin) OR (ALL,FT(hexachloro) AND ALL,FT(hexahydro)) OR ALL,FT(dimethanonaphthalene) OR (ALL,FT(hexachloro) AND ALL,FT(hexahydro)) OR ALL,FT(aldrex) OR ALL,FT("compound 118") OR ALL,FT(octalene) OR ALL,FT(photoaldrin) OR (ALL,FT(alpha) AND ALL,FT(hexachlorocyclohexane)) OR ALL,FT("alpha HCH") OR ALL,FT(HCH) OR (ALL,FT(benzene) AND ALL,FT(hexachloride)) OR (ALL,FT(alpha) AND ALL,FT(benzenehexachlor)) OR (ALL,FT(alpha) AND ALL,FT(benzenehexachloride)) OR ALL,FT("alpha bhc") OR ALL,FT("alpha hch") OR (ALL,FT(beta) AND ALL,FT(hexachlorocyclohexane)) OR ALL,FT(cyclohexane) OR (ALL,FT(hexachloro) AND ALL,FT(beta)) OR (ALL,FT(beta) AND ALL,FT(hexachlorobenzene)) OR (ALL,FT(beta) AND ALL,FT(HCH)) OR ALL,FT("beta Lindane") OR (ALL,FT(beta) AND ALL,FT(Lindane)) OR ALL,FT("beta benzene") OR (ALL,FT(beta) AND ALL,FT(benzene)) OR ALL,FT(hexachloride) OR ALL,FT("beta hexachloran") OR (ALL,FT(beta) AND ALL,FT(hexachloran)) OR ALL,FT("beta 666") OR ALL,FT("epsilon HCH") OR (ALL,FT(beta) AND ALL,FT(benzenehexachloride)) OR ALL,FT("beta bhc") OR ALL,FT("beta hch") OR ALL,FT(campheclor) OR ALL,FT(alltox) OR ALL,FT(camfeclor) OR ALL,FT(camphechlor) OR (ALL,FT(camphene) AND ALL,FT(chlorinated)) OR ALL,FT("chlorinated camphene") OR ALL,FT("chlorphen clorphen") OR ALL,FT(estonox) OR ALL,FT(geniphene) OR ALL,FT(penfene) OR ALL,FT(phenacide) OR ALL,FT(phenatox) OR ALL,FT(phenphene) OR ALL,FT(phenphere) OR ALL,FT(pinetox) OR ALL,FT(polychlorocamphene) OR ALL,FT(toxakil) OR ALL,FT(toxaphen) OR ALL,FT(toxaphene) OR ALL,FT(chlordane) OR ALL,FT("gamma Chlordane") OR ALL,FT(Chlordane) OR (ALL,FT(octachloro) AND ALL,FT(alpha)) OR (ALL,FT(alpha) AND ALL,FT(hexahydro)) OR ALL,FT(methanoindene) OR (ALL,FT(octachloro) AND ALL,FT(alpha)) OR (ALL,FT(alpha) AND ALL,FT(tetrahydro)) OR ALL,FT(methanoindan) OR (ALL,FT(octachloro) AND ALL,FT(methane)) OR (ALL,FT(alpha) AND ALL,FT(tetrahydroindane)) OR (ALL,FT(alpha) AND ALL,FT(chlordane)) OR ALL,FT(alphachlordane) OR ALL,FT("cd 68") OR ALL,FT(cd68) OR ALL,FT(chlordane) OR (ALL,FT(cis) AND ALL,FT(chlordane)) OR (ALL,FT(cis) AND ALL,FT(photochlordane)) OR ALL,FT("compound 1068") OR ALL,FT("hcs 3260") OR ALL,FT("m 410") OR ALL,FT("octa klor") OR (ALL,FT(cis) AND ALL,FT(chlordane)) OR ALL,FT(photochlordane) OR ALL,FT(toxichlor) OR (ALL,FT(trans) AND ALL,FT(chlordane)) OR ALL,FT("velsicol 1068") OR ALL,FT(wydane) OR ALL,FT(chlordecone) OR ALL,FT(Kepone) OR ALL,FT(decachlorooctahydro) OR ALL,FT("metheno cyclobuta") OR ALL,FT("pentalen one") OR ALL,FT("cg 1189") OR ALL,FT(cg1189) OR ALL,FT(kepone) OR ALL,FT(chlorphenotane) OR ALL,FT(chlorobenzene) OR ALL,FT(trichloroethylidene) OR (ALL,FT(chlorophenyl) AND ALL,FT(trichloroethane)) OR ALL,FT(trichloroethane) OR ALL,FT(trichloroethene) OR ALL,FT(benzochloryl) OR ALL,FT(chlorophenoltane) OR ALL,FT(chlorophenotane) OR ALL,FT(chlorophenothane) OR ALL,FT(chlorphenethanum) OR ALL,FT(chlorphenotan) OR ALL,FT(chlorphenothane) OR ALL,FT(chlorphenothanum) OR ALL,FT(clofenotan) OR ALL,FT(clofenotane) OR ALL,FT(d.d.t) OR ALL,FT(DDT) OR ALL,FT("ddt residue") OR ALL,FT(detane) OR ALL,FT(dichlordiphenyltrichlormethylmethane) OR ALL,FT(dichlordiphenyltrichloroethane) OR ALL,FT(dichlorodiphenyltrichloroethane) OR ALL,FT(dicophane) OR ALL,FT(dodat) OR ALL,FT(esoderm) OR ALL,FT(estonate) OR ALL,FT(gesapon) OR ALL,FT(gesarex) OR ALL,FT(gesarol) OR ALL,FT(lentinol) OR ALL,FT(neocide) OR ALL,FT("para ddt") OR ALL,FT(parachlorocide) OR ALL,FT(pentachlorin) OR ALL,FT(penticidum) OR ALL,FT(pestanal) OR ALL,FT(suleo) OR ALL,FT(ethane) OR ALL,FT(trichomon) OR ALL,FT(clofentezine) OR ALL,FT(bisclofentezin) OR (ALL,FT(chlorophenyl) AND ALL,FT(tetrazine)) OR ALL,FT(tetrazine) OR ALL,FT(dieldrin) OR ALL,FT("Alvit 55") OR ALL,FT(Alvit55) OR (ALL,FT(hexachloro) AND ALL,FT(epoxy)) OR ALL,FT("octahydro endo") OR ALL,FT("10 para") OR ALL,FT("meta dieldrin") OR ALL,FT("compound 497") OR (ALL,FT(dieldrin) AND ALL,FT(abavit)) OR ALL,FT(dieldrine) OR ALL,FT(heod) OR ALL,FT(photodieldrin) OR ALL,FT(endosulfan) OR ALL,FT(Thiodan) OR ALL,FT(Thiodon) OR (ALL,FT(beta) AND ALL,FT(Endosulfan)) OR ALL,FT(Thiotox) OR (ALL,FT(alpha) AND ALL,FT(Endosulfan)) OR (ALL,FT(hexachloronorbornene) AND ALL,FT(sulfite)) OR (ALL,FT(hydroxymethyl) AND ALL,FT(hexachloronorbornenesulfite)) OR (ALL,FT(hydroxymethyl) AND ALL,FT(hexachlorobicyclo)) OR (ALL,FT(ene) AND ALL,FT(sulfite)) OR (ALL,FT(hydroxymethyl) AND ALL,FT(hexachloronorcamphene)) OR (ALL,FT(alpha) AND ALL,FT(benzoepin)) OR (ALL,FT(alpha) AND ALL,FT(endosulfan)) OR (ALL,FT(alpha) AND ALL,FT(endosulfane)) OR ALL,FT(benzoepin) OR ALL,FT(beosit) OR (ALL,FT(beta) AND ALL,FT(benzoepin)) OR (ALL,FT(beta) AND ALL,FT(endosulfan)) OR (ALL,FT(beta) AND ALL,FT(endosulfane)) OR ALL,FT(chlorothiepine) OR ALL,FT(chlorthiapinum) OR ALL,FT(chlorthiepin) OR ALL,FT(cyclodan) OR ALL,FT(endogan) OR ALL,FT(ensawan) OR ALL,FT("fmc 5462") OR ALL,FT(fmc5462) OR (ALL,FT(hexachlorohexahydro) AND ALL,FT(methano)) OR (ALL,FT(benzodioxathiepine) AND ALL,FT(oxide)) OR ALL,FT(hexachloronorbornene) OR (ALL,FT(oxymethylene) AND ALL,FT(sulfite)) OR ALL,FT(malix) OR ALL,FT(sialan) OR ALL,FT(thifor) OR ALL,FT(thimul) OR ALL,FT(thiodan) OR ALL,FT(thionex) OR ALL,FT(thiotox) OR ALL,FT(thyodan) OR ALL,FT(thyonex) OR ALL,FT(tiodan) OR ALL,FT(endrin) OR ALL,FT(Hexadrin) OR ALL,FT("compound 269") OR (ALL,FT(compound) AND ALL,FT(dieldrin)) OR ALL,FT(hexadrin) OR ALL,FT(heptachlor) OR (ALL,FT(heptachloro) AND ALL,FT(tetrahydro)) OR ALL,FT(methanoindene) OR ALL,FT(heptachlore) OR ALL,FT(heptachloro) OR (ALL,FT(tetrahydro) AND ALL,FT(methanoindene)) OR ALL,FT(photoheptachlor) OR (ALL,FT(heptachlor) AND ALL,FT(epoxide)) OR (ALL,FT(Epoxide) AND ALL,FT(Heptachlor)) OR ALL,FT(Heptachlorepoxide) OR ALL,FT(isobenzan) OR (ALL,FT(octachloro) AND ALL,FT(endomethylene)) OR ALL,FT(tetrahydrophthalan) OR ALL,FT(octachloro) OR (ALL,FT(hexahydro) AND ALL,FT(methanoisobenzofuran)) OR (ALL,FT(hexahydro) AND ALL,FT(methanoisobenzofuran)) OR (ALL,FT(octachloro) AND ALL,FT(hexahydro)) OR ALL,FT(methanoisobenzofuran) OR (ALL,FT(betahydro) AND ALL,FT(methanophthalan)) OR ALL,FT(izobenzan) OR ALL,FT(omtan) OR ALL,FT(ontan) OR ALL,FT("r 6700") OR ALL,FT("sd 4402") OR ALL,FT(telodrin) OR ALL,FT(lindane) OR ALL,FT(Hexachlorane) OR (ALL,FT(Benzene) AND ALL,FT(Hexachloride)) OR (ALL,FT(Hexachloride) AND ALL,FT(Benzene)) OR (ALL,FT(Epsilon) AND ALL,FT(hexachlorocyclohexane)) OR (ALL,FT(Epsilon) AND ALL,FT(hexachlorocyclohexane)) OR (ALL,FT(Zeta) AND ALL,FT(hexachlorocyclohexane)) OR (ALL,FT(Zeta) AND ALL,FT(hexachlorocyclohexane)) OR ALL,FT(Lindane) OR (ALL,FT(Benzene) AND ALL,FT(Hexachloride)) OR (ALL,FT(Hexachloride) AND ALL,FT(gamma)) OR (ALL,FT(gamma) AND ALL,FT(Benzene)) OR ALL,FT("Gamma 666") OR ALL,FT(Gammexane) OR ALL,FT("gamma HCH") OR (ALL,FT(gamma) AND ALL,FT(Hexachlorocyclohexane)) OR ALL,FT("BHC Insecticide") OR ALL,FT("Insecticide BHC") OR ALL,FT("PMS Lindane") OR (ALL,FT(PMS) AND ALL,FT(Lindane)) OR ALL,FT(Kwell) OR ALL,FT(Scabecid) OR ALL,FT(Jacutin) OR ALL,FT(Scabene) OR ALL,FT(Tetocid) OR ALL,FT(Scabisan) OR (ALL,FT(Eta) AND ALL,FT(hexachlorocyclohexane)) OR (ALL,FT(Eta) AND ALL,FT(hexachlorocyclohexane)) OR ALL,FT(Delitex) OR ALL,FT(acaricida) OR ALL,FT(aparasin) OR ALL,FT(aphthiria) OR ALL,FT(aphtiria) OR ALL,FT(atan) OR ALL,FT("battle bhc") OR ALL,FT("benhex cream") OR ALL,FT(bhc) OR ALL,FT(bicide) OR ALL,FT(chloresene) OR ALL,FT(dagicide) OR ALL,FT(davesol) OR ALL,FT(delice) OR ALL,FT(delitex) OR ALL,FT(devoran) OR ALL,FT(elentol) OR ALL,FT(entomoxan) OR ALL,FT(forlin) OR ALL,FT(gambex) OR ALL,FT(gamene) OR ALL,FT(gamiso) OR (ALL,FT(gamma) AND ALL,FT(hexachlorocyclohexane)) OR ALL,FT("gamma 666") OR ALL,FT("gamma benzene") OR ALL,FT("gamma bhc") OR ALL,FT("gamma hch") OR (ALL,FT(gamma) AND ALL,FT(hexachlorocyclohexane)) OR ALL,FT(gammahexachlorcyclohexane) OR ALL,FT(gammexane) OR ALL,FT(geksan) OR ALL,FT(gexane) OR ALL,FT(hch) OR ALL,FT(herklin) OR ALL,FT(hexachloran) OR ALL,FT(hexachlorane) OR ALL,FT(hexaverm) OR ALL,FT(hexchloran) OR ALL,FT(hexicide) OR ALL,FT(hexit) OR ALL,FT(hexyclan) OR ALL,FT(hisdane) OR ALL,FT(jacuta) OR ALL,FT(jacutin) OR ALL,FT(kwell) OR ALL,FT("kwell lotion") OR ALL,FT("kwell shampoo") OR ALL,FT(kwellada) OR ALL,FT(lencid) OR ALL,FT(lindactone) OR ALL,FT(lindagam) OR ALL,FT(linden) OR ALL,FT("linden lotion") OR ALL,FT(lorexane) OR (ALL,FT(neo) AND ALL,FT(scabicidol)) OR ALL,FT(nourycid) OR ALL,FT("pms lindane") OR ALL,FT(quellada) OR (ALL,FT(quellada) AND ALL,FT(cream)) OR ALL,FT("quellada crème") OR ALL,FT("quellada head") OR ALL,FT("lice treatment") OR ALL,FT("quellada lotion") OR ALL,FT("quellada h") OR ALL,FT(sarconyl) OR ALL,FT(scabecid) OR ALL,FT(scabene) OR ALL,FT("scabene lotion") OR ALL,FT(scabexyl) OR ALL,FT(scabi) OR ALL,FT(scabien) OR ALL,FT(scabisan) OR ALL,FT(streunex) OR ALL,FT("tigal f") OR ALL,FT("tri 6") OR ALL,FT(trisix) OR ALL,FT(varsan) OR ALL,FT(vermexane) OR ALL,FT(veticide) OR ALL,FT(methoxychlor) OR ALL,FT(DMDT) OR (ALL,FT(Dianisyl) AND ALL,FT(Trichloroethane)) OR (ALL,FT(Trichloroethane) AND ALL,FT(Dianisyl)) OR ALL,FT(Metox) OR (ALL,FT(methoxyphenyl) AND ALL,FT(ethane)) OR (ALL,FT(methoxyphenyl) AND ALL,FT(trichloroethane)) OR (ALL,FT(methoxyphenyl) AND ALL,FT(trichloroethane)) OR (ALL,FT(anisyl) AND ALL,FT(trichloroethane)) OR ALL,FT(marlate) OR (ALL,FT(methoxy) AND ALL,FT(chlor)) OR (ALL,FT(methoxy) AND ALL,FT(ddt)) OR ALL,FT(methoxyclor) OR ALL,FT(mirex) OR (ALL,FT(dodecachlorooctahydro) AND ALL,FT(metheno)) OR (ALL,FT(cyclobuta) AND ALL,FT(pentalene)) OR ALL,FT(mirax) OR ALL,FT(perchloropentacyclodecane) OR ALL,FT(nonachlor) OR (ALL,FT(nonachloro) AND ALL,FT(tetrahydro)) OR ALL,FT(methanoindan) OR (ALL,FT(nonachlor) AND ALL,FT(isomer)) OR ALL,FT(oxychlordane) OR (ALL,FT(beta) AND ALL,FT(octachloro)) OR (ALL,FT(alpha) AND ALL,FT(epoxy)) OR (ALL,FT(alpha) AND ALL,FT(tetrahydro)) OR ALL,FT(oxychlordan) OR ALL,FT(photomirex) OR ALL,FT(monohydromirex) OR ALL,FT(hydromirex) OR ALL,FT("mirex hydro") OR (ALL,FT(mirex) AND ALL,FT(hydro)) OR ALL,FT("mirex monohydro") OR (ALL,FT(mirex) AND ALL,FT(monohydro)) AND YP(19900101-20230901)
